# Supplementary material for: Derivation of embryonic stem cells from cloned blastocysts using improved somatic cell nuclear transfer in common marmosets
Source: Stem Cell Reports. 2025 Nov 13;20(12):102710. doi: 10.1016/j.stemcr.2025.102710 (PMC12744851; doi:10.1016/j.stemcr.2025.102710)
Supplement: Document S1. Figures S1 and S2, Tables S1, S4, and S5, and supplemental methods [file mmc1.pdf]

Stem Cell Reports, Volume *20*

Supplemental Information

Derivation of embryonic stem cells from cloned blastocysts using improved somatic cell nuclear transfer in common marmosets

Shogo Matoba, Yoko Kurotaki, Satoshi Funaya, Yuko Yamada, Narumi Ogonuki, Haruka Shinohara, Masafumi Yamamoto, Nao Yoneda, Takaya Homma, Yuichiro Higuchi, Erika Sasaki, and Atsuo Ogura

Supplemental Figures

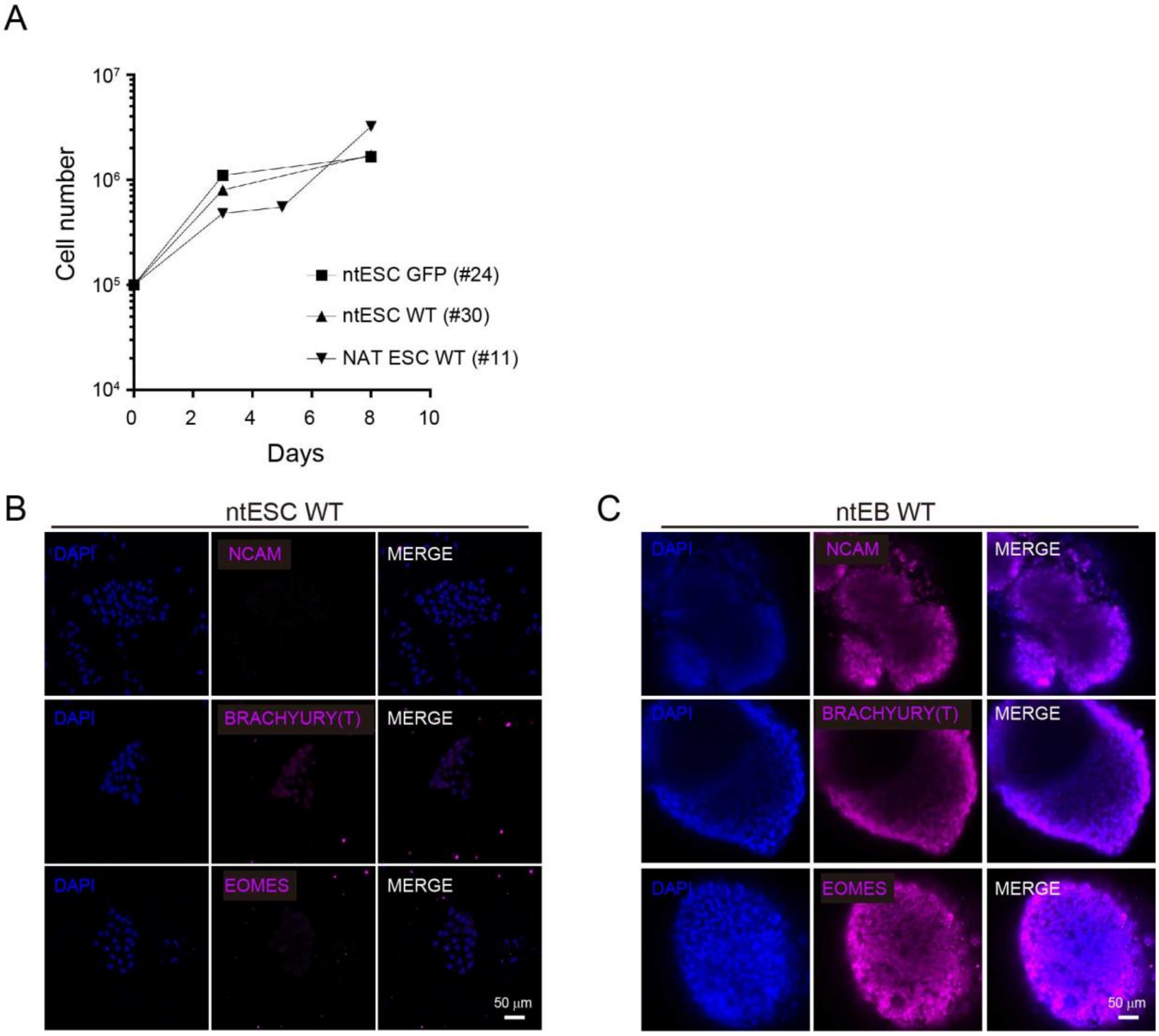

Figure S1. Proliferation and in vitro differentiation of ntESCs to form ntEBs, related to Figure 5

(A) Proliferation of ESCs and ntESCs. (B-C) Immunostaining (B) ntESCs and (C) ntEBs of WT #61 line for markers of three germ layers.

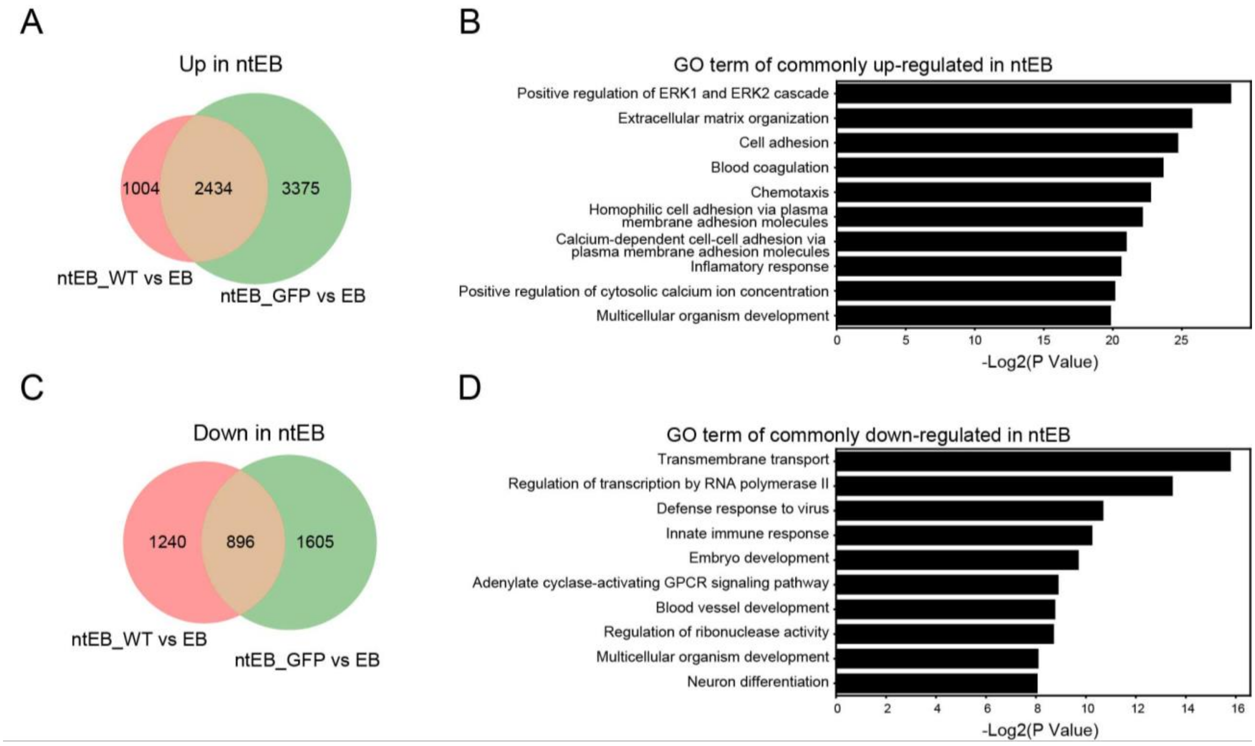

Figure S2. Abnormally expressed genes in ntEB, related to Figure 6
(A) Venn diagram showing commonly upregulated genes in ntEB.
(B) GO term enriched in the commonly upregulated genes in ntEB.
(C) Venn diagram showing commonly downregulated genes in ntEB.
(D) GO term enriched in the commonly downregulated genes in ntEB.

Supplemental Tables

Table S1. List of established ntESCs in this study, related to Figure 3

Name of ESC	Genotype	Manipulation	Sex of donor cell	Karyotype analysis		
				Passage number	Mode number	Karyotype
ntESC WT #30	WT	SCNT	XY	6	46 (84%)	Normal 46, XY [8/8]
ntESC WT #53	WT	SCNT	XY	11	46 (90%)	Normal 46, XY [5/5]
ntESC WT #61	WT	SCNT	XY	10	46 (90%)	Normal 46, XY [8/8]
ntESC GFP #24	CAG-GFP	SCNT	XX	5	46 (94%)	Normal 46, XX [5/5]
ntESC GFP #50	CAG-GFP	SCNT	XX	6	46 (89%)	Normal 46, XX [5/5]
ntESC GFP #54	CAG-GFP	SCNT	XX	6	46 (98%)	Normal 46, XX [5/5]
				11	46 (98%)	Abnormal 46, XX, t(7p;15q) [5/5]
ntESC GFP #55	CAG-GFP	SCNT	XX	7	46 (80%)	Normal 46, XX [5/5]
ESC NAT #11	WT	NAT	XY	7	46 (58%)	Normal 46, XY [11/11]
ESC NAT #13	WT	NAT	XX	18	46 (88%)	Normal 46, XX [7/7]
ESC NAT #37	WT	NAT	XY	7	46 (86%)	Normal 46, XY [6/6]
ESC IVF #15	WT	IVF	XX	14	46 (86%)	Normal 46, XX [7/7]
ESC IVF #17	WT	IVF	XY	21	46 (66%)	Normal 46 XY [6/6]

*NAT and IVF derived ESCs were reported in Kishimoto et al. 2021.

Table S2. RNA-sequencing data comparing control ESC and ntESC, related to Figures 4, 5 and 6 (see Excel file)

Table S3. RNA-sequencing data comparing control EB and ntEB, related to Figures 5 and S2 (see Excel file).

Table S4. List of primers used for microsatellite marker analysis, related to Figure 3

Type	Name of primer	Dye	Sequence
Dye-labeled primer	2463P-TH	FAM	GCACAGGCAGATTCAAGACAACTC
	CJ060	PET	TGCTCTAGAGGTTCCACTCTG
	CJ077	PET	ATTCCATTCTGGGCAGCAAG
	CJ081	VIC	TTCCCCTCTCTTTCAGACACA
	CJ103	NED	CCCTTTCCTGCTAATTCACAGAAG
	CJ187	FAM	TGGAAGAACTTTCTGCCAAACC
	CJ003	NED	AGATGTGGCAGTTGTCTTGG
	CJ083	VIC	TTGTACCCTTTTGCTTGCAG
	CJ091	FAM	CCTGCACCCGTAAATAGGTTC
	CJ146	PET	CTTAATTCTGCCACAGTAGCAC
	CjX	FAM	CCAGCTACTCATGAAGTTGAG
	CjY	VIC	CATTGTGCCACTGCAGAATTA
Non-labeled primer	2463P-TH-NL	None	CCAAGACCTCAGGGAGGTAGTAGG
	CJ060-NL	None	GGCATGTTACCTAACCTCTCTG
	CJ077-NL	None	CCTCCCATACTACAGATGAGGA
	CJ081-NL	None	CACCTCCTCTTCAAGTAAACACC
	CJ103-NL	None	CTGGGTAACAAGAGTGAAACTCC
	CJ187-NL	None	GCTTGTTCAGGCAGACTGAC
	CJ003-NL	None	TCTCTGCCATAGTGACCTCT
	CJ083-NL	None	TTCCTTCTTTTGGGGAGTGT
	CJ091-NL	None	CATCCTGGGCAACAAGAGTG
	CJ146-NL	None	GAGAGTCCCTAAATGCAAGGA
	CjX-NL	None	CTCTTCATATTCTTATGATGTGTGC
	CjY-NL	None	GAAGGTAATCCATTTCACTGAGC

Table S5. List of antibodies used for immunofluorescence staining, related to Figures 1 and 4

Antibody name	Dilution	Source animal	Catalog number	Clone ID	Company
anti-H3K9me3	1:500	Rabbit	ab8898		Abcam
anti-NANOG	1:200	Mouse	4893	1E6C4	Cell Signaling Technology
anti-CDX2	1:400	Rabbit	3977		Cell Signaling Technology
anti-GFP	1:100	Rabbit	ab183734		Abcam
anti-OCT3/4	1:50	Mouse	sc-5279	C-10	Santa Cruz Biotechnology
anti-SOX2	1:200	Mouse	4900	L1D6A2	Cell Signaling Technology
anti-LIN28A	1:200	Rabbit	3978		Cell Signaling Technology
anti-SSEA4	1:200	Mouse	sc-21704	813-70	Santa Cruz Biotechnology
anti-TRA-1-60	1:100	Mouse	MAB4360	TRA-1-60	Thermo Fisher Scientific
anti-TRA-1-81	1:100	Mouse	MAB4381	TRA-1-81	Merck Millipore
anti-UTF1	1:100	Mouse	MAB4337	5G10.2	Merck Millipore
anti-NCAM-L1	1:200	Rabbit	89861	D5N9S	Cell Signaling Technology
anti-BRACHYURY (T)	1:200	Rabbit	ab20680		Abcam
anti-EOMES	1:100	Rabbit	ab23345		Abcam

Supplemental methods

Oocyte collection and in vitro maturation

Oocyte retrieval was performed as previously described (Kurotaki and Sasaki, 2017; Sato et al., 2016; Takahashi et al., 2014; Tomioka et al., 2012). To synchronize the estrous cycle, female common marmosets were injected intramuscularly with 0.8 µg of prostaglandin F2α (MSD Animal Health, USA) to induce luteolysis. The next day, serum progesterone concentration was confirmed to have decreased, and ovarian stimulation was initiated. Recombinant follicle-stimulating hormone (rFSH; 25 IU/head; Gonal-f 150IU, Merck, Germany) was administered intramuscularly every second day for 9 days. On the evening of day 10, human chorionic gonadotropin (hCG; 75 IU/head; ASKA Pharmaceutical, Japan) was injected. Oocyte pick-up (OPU) was performed by laparotomy 18 hours after hCG injection.

For pre-anesthesia, animals were given 0.15 ml of an MMB cocktail (medetomidine 0.04 mg/kg, midazolam 0.4 mg/kg, butorphanol 0.4 mg/kg and saline at a 1:2:2:5 ratio: Dexmedetomidine, Sandoz, Japan; Midazolam, Teva Takeda Pharma, Japan; Vetorphale, Meiji Seika Pharma, Japan; and saline, Otsuka Pharmaceutical, Japan). To prevent infection and dehydration, ampicillin (15 mg/kg, Fujita Pharmaceutical, Japan) and an electrolyte solution (KN No. 1, Otsuka Pharmaceutical) were administered intramuscularly. Anesthesia was maintained using 1.0%–3.0% isoflurane with a face mask. After oocyte retrieval, the animal's abdomen was sutured, and the animal was rested for at least 2 months before the next OPU. Atipamezole (0.20 mg/kg, Nippon Zenyaku Kogyo, Japan) was administered for recovery. For post-operative care, ketoprofen (1.2 mg/kg, Nissin Pharmaceutical, Japan) and ampicillin (15 mg/kg) were administered daily for three days.

Oocytes were aspirated from ovarian follicles using a 25-gauge needle and cultured in porcine oocyte medium (POM) (IFP1010P, Research Institute for the Functional Peptides, Japan). The retrieved GV oocytes were matured in vitro in POM supplemented with 0.15 IU/mL hFSH, 10 IU/mL hCG and 5% FBS for 24 hours under controlled conditions of 37.5 °C in a humidified atmosphere containing 5% CO_2 and 5% O_2 in air. Cumulus cells were removed by hyaluronidase treatment, and MII oocytes were selected for SCNT.

Preparation of donor cells

Somatic cells used for nuclear transfer included cumulus cells and fibroblasts. Cumulus cells were collected by enzymatic dissociation of cumulus-oocyte complexes obtained from in vitro-matured oocytes derived from WT female marmosets.

Fibroblasts were isolated from ear skin biopsies of WT and GFP-transgenic marmosets using standard tissue digestion protocols. Cells were cultured for several days and frozen at early passages (P3–P6). For SCNT, fibroblasts were thawed and seeded into 60-mm culture dishes (3010-060, AGC Techno Glass, Japan) 7 days prior to use. After reaching confluency, cells were maintained for several days to synchronize in the G1/G0 phase.

Immunofluorescence staining

SCNT embryos, ntESCs, or ntEBs were fixed with 4% PFA in PBS for 30 minutes at room temperature, washed with PBS, and permeabilized with 0.5% Triton X-100 for 10 minutes. After blocking with 5% FBS in PBS for 30 minutes, embryos or cells were incubated overnight at 4 °C with primary antibodies (Table S5). After washing, samples were incubated with Alexa Fluor 555-conjugated donkey anti-rabbit IgG, Alexa Fluor 647-conjugated donkey anti-mouse, or anti-rabbit IgG secondary antibodies (Thermo Fisher Scientific) for 30 minutes at room temperature. Nuclei were stained with DAPI (P36931, Invitrogen, USA) or Hoechst 33342 (346-07951, DOJINDO). Samples were visualized using an IX71 fluorescence microscope (Olympus, Japan) or LSM880 confocal microscope (Zeiss, Germany).

Supplemental references

Kurotaki, Y., and Sasaki, E. (2017). Practical reproductive techniques for the common marmoset. J. Mamm. Ova Res. *34*, 3–12. 10.1274/032.034.0103.

Sato, K., Oiwa, R., Kumita, W., Henry, R., Sakuma, T., Ito, R., Nozu, R., Inoue, T., Katano, I., Sato, K., et al. (2016). Generation of a nonhuman primate model of severe combined immunodeficiency using highly efficient genome editing. Cell Stem Cell *19*, 127–138. 10.1016/j.stem.2016.06.003.

Takahashi, T., Hanazawa, K., Inoue, T., Sato, K., Sedohara, A., Okahara, J., Suemizu, H., Yagihashi, C., Yamamoto, M., Eto, T., et al. (2014). Birth of healthy offspring following ICSI in in vitro-matured common marmoset (Callithrix jacchus) oocytes. PLoS One *9*, e95560. 10.1371/journal.pone.0095560.

Tomioka, I., Takahashi, T., Shimada, A., Yoshioka, K., and Sasaki, E. (2012). Birth of common marmoset (Callithrix jacchus) offspring derived from in vitro-matured oocytes in chemically defined medium. Theriogenology *78*, 1487–1493. 10.1016/j.theriogenology.2012.06.024.
