# Supplementary material for: Derivation of embryonic stem cells from cloned blastocysts using improved somatic cell nuclear transfer in common marmosets
Source: Stem Cell Reports. 2025 Nov 13;20(12):102710. doi: 10.1016/j.stemcr.2025.102710 (PMC12744851; doi:10.1016/j.stemcr.2025.102710)
Supplement: Document S2. Article plus supplemental information [file mmc4.pdf]

STEM CELL REPORTS
Article
OPEN ACCESS

Derivation of embryonic stem cells from cloned blastocysts using improved somatic cell nuclear transfer in common marmosets

Shogo Matoba,[1,2,10,11,*] Yoko Kurotaki,[1,3,10] Satoshi Funaya,[1] Yuko Yamada,[4] Narumi Ogonuki,[1] Haruka Shinohara,[4] Masafumi Yamamoto,[5] Nao Yoneda,[6] Takaya Homma,[6] Yuichiro Higuchi,[6] Erika Sasaki,[4,7,*] and Atsuo Ogura[1,8,9,*]

[1]Integrative Developmental Engineering Division, RIKEN Bioresource Research Center, Tsukuba, Ibaraki 305-0074, Japan
[2]Cooperative Division of Veterinary Sciences, Tokyo University of Agriculture and Technology, Fuchu, Tokyo 183-8509, Japan
[3]Department of Development Research Translational Research Division, Central Institute for Experimental Medicine and Life Science, Kawasaki, Kanagawa 210-0821, Japan
[4]Division of Advanced Physiology, Central Institute for Experimental Medicine and Life Science, Kawasaki, Kanagawa 210-0821, Japan
[5]ICLAS Monitoring Center, Central Institute for Experimental Medicine and Life Science, Kawasaki, Kanagawa 210-0821, Japan
[6]Liver Engineering Laboratory, Department of Research for Humanized Model, Central Institute for Experimental Medicine and Life Science, Kawasaki, Kanagawa 210-0821, Japan
[7]Laboratory for Proteolytic Neuroscience, RIKEN Center for Brain Science, Wako, Saitama 351-0198, Japan
[8]Graduate School of Life and Environmental Sciences, University of Tsukuba, Tsukuba, Ibaraki 305-8572, Japan
[9]The Center for Disease Biology and Integrative Medicine, Faculty of Medicine, University of Tokyo, Bunkyo-ku, Tokyo 113-0033, Japan
[10]These authors contributed equally
[11]Lead contact
*Correspondence: shogo.matoba@riken.jp (S.M.), esasaki@ciea.or.jp (E.S.), atsuo.ogura@riken.jp (A.O.)
https://doi.org/10.1016/j.stemcr.2025.102710

SUMMARY

The common marmoset (*Callithrix jacchus*) is a genetically modifiable non-human primate increasingly used in biomedical research. Here, we established a method for deriving embryonic stem cells (ESCs) from blastocysts generated by somatic cell nuclear transfer (SCNT) in the marmoset. Injection of histone demethylase *Kdm4d* mRNA enabled efficient reprogramming of somatic nuclei, allowing blastocyst formation in 14.5% from fibroblasts. Combining this method with a G9a/EHMT2 histone methyltransferase inhibitor improved blastocyst quality and allowed derivation of nuclear transfer ESCs (ntESCs), including wild-type and GFP-transgenic lines. These ntESCs exhibited normal karyotypes and pluripotency. Nuclear and mitochondrial DNA analyses confirmed their nuclear donor origin and cytoplasmic inheritance from recipient oocytes. Transcriptome analysis identified abnormally expressed genes in ntESCs present in a line-dependent and independent manner, suggesting partial reprogramming resistance. Our study establishes a marmoset SCNT method enabling derivation of ntESCs and provides a new platform for preserving and engineering marmoset genetic resources.

INTRODUCTION

The common marmoset (*Callithrix jacchus*) has become an increasingly important non-human primate model in biomedical research because of its small body size, rapid reproductive cycle, and expanding tractability to genetic manipulation (Inoue et al., 2023; Kurotaki and Sasaki, 2017; Mansfield, 2003). Unlike macaques, marmosets often produce multiple neonates in the litter and reach sexual maturity quickly, making them well suited for transgenerational studies and disease modeling. Transgenic and genome-edited marmosets have been produced using approaches such as lentivirus vector-based transgenesis (Heide et al., 2020; Sasaki et al., 2009) or genome editing in zygotes or early embryos (Abe et al., 2021; Sato et al., 2016, 2024). These approaches have generated novel genetic models suitable for neuroscience studies (Sato and Sasaki, 2018) and those on evolution (Heide et al., 2020). Even so, the overall efficiency of generating and propagating genetically modified lines remains low, and robust technologies for preserving or expanding specific genotypes, and particularly from valuable or difficult-breed founders, are still underdeveloped in this species.

Somatic cell nuclear transfer (SCNT) is a powerful technique that allows the reprogramming of a somatic nucleus into a totipotent state by transferring it into an enucleated oocyte (Matoba and Zhang, 2018). SCNT has been successfully applied to generate cloned animals in a wide variety of mammalian species, including mice, cows, pigs, and cynomolgus monkeys (Liu et al., 2018; Matoba and Zhang, 2018; Ogura et al., 2021). In the common marmoset, however, reconstructed SCNT embryos have shown poor developmental competence, with most embryos arresting development before the morula stage and no reports of blastocyst formation or viable offspring to date (Sotomaru et al., 2009). Work in mice and other species has established that persistent epigenetic barriers—most notably donor cell-derived repressive histone mark H3K9me3, DNA methylation, and loss of H3K27me3-mediated imprinting—impede zygotic genome activation (ZGA) and downstream development in SCNT embryos (Liu et al., 2016; Matoba et al., 2014; Xu et al., 2023).

© 2025 The Author(s).
Published by Elsevier Inc. on behalf of International Society for Stem Cell Research.
This is an open access article under the CC BY license (http://creativecommons.org/licenses/by/4.0/).

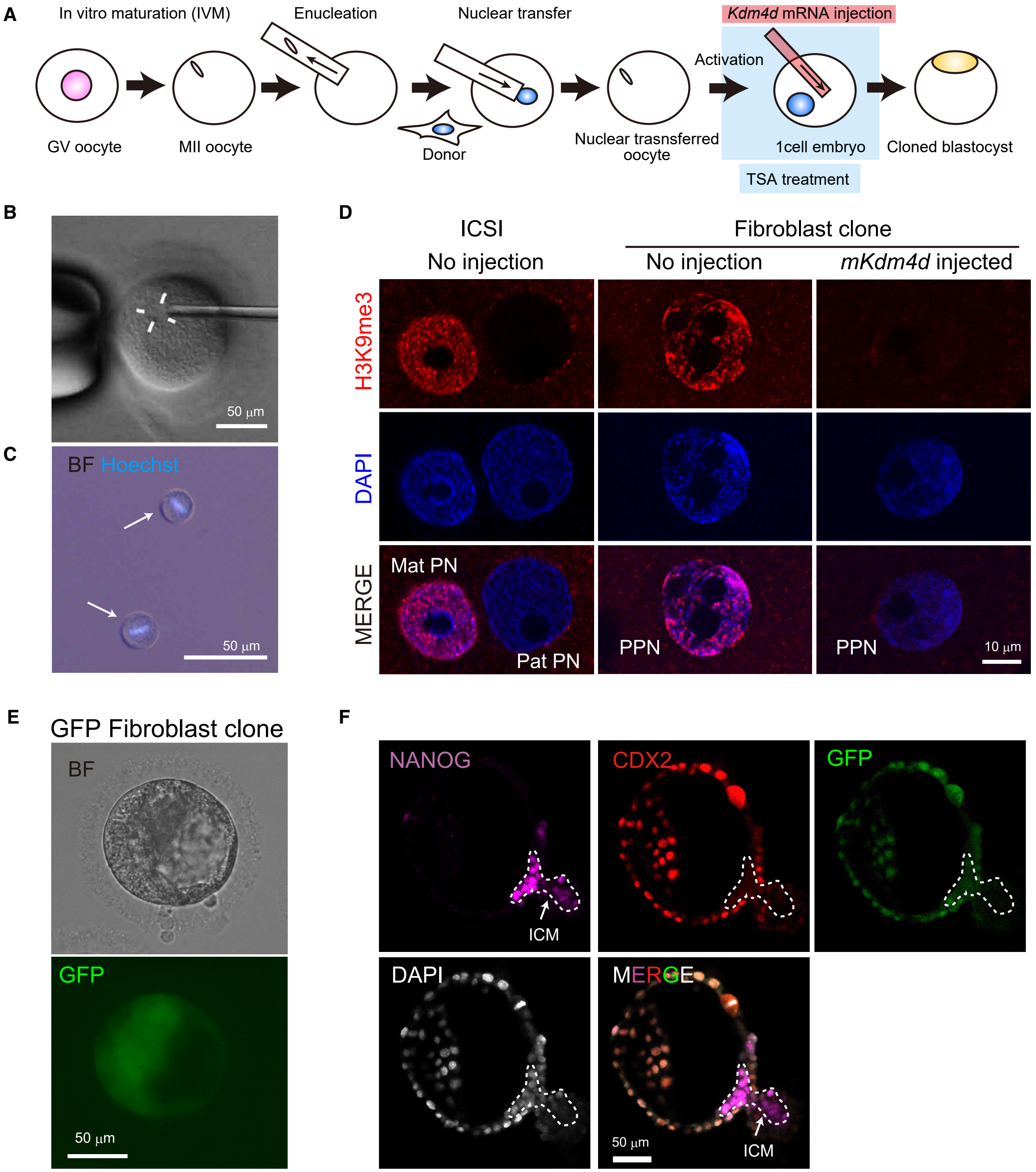

Figure 1. Establishment of basic procedures of SCNT in marmosets

(A) Schematic overview of the SCNT procedure. Germinal vesicle-stage oocytes were matured *in vitro*, enucleated, and fused or injected with donor somatic cells. Reconstructed oocytes were activated to initiate development. The activated SCNT embryos were treated with TSA for 8 h and injected with mouse *Kdm4d* mRNA at the 1-cell stage.

(B) Enucleation step of marmoset MII oocyte. The metaphase spindle is marked by white lines. Scale bar, 50 μm.

(legend continued on next page)

Several epigenetic interventions partially overcome these barriers. Expression of the H3K9me3 demethylase *Kdm4d* markedly improves the development of mouse, monkey, and human SCNT embryos (Chung et al., 2015; Liu et al., 2018, 2019; Matoba et al., 2014), while histone deacetylase inhibitors such as trichostatin A (TSA) or scriptaid enhance transcriptional reactivation and survival after nuclear transfer (Kishigami et al., 2006; Van Thuan et al., 2009). Use of other compounds, such as latrunculin A, which inhibits actin polymerization (Himaki et al., 2010; Terashita et al., 2012), and vitamin C, known to promote DNA demethylation (Blaschke et al., 2013), has also been reported to improve the embryonic development of SCNT embryos in various mammalian models (Fang et al., 2022; Miyamoto et al., 2017). More recently, our group also demonstrated that inhibition of the histone methyltransferase G9a/EHMT2 can further facilitate transcriptional reactivation in mouse SCNT embryos, primarily through reduction of the repressive H3K9me3 mark (Matoba et al., 2024). Whether these epigenetic barriers are conserved in the marmoset and whether overcoming them would similarly improve SCNT embryo development in this species remains unclear.

Here, we applied a combined epigenetic enhancement strategy—TSA exposure, *Kdm4d* mRNA injection, and G9a inhibition—to improve nuclear reprogramming after SCNT in the common marmoset. Using this approach, we obtained blastocysts at reproducible frequencies from fibroblast donors and, importantly, established multiple stable nuclear transfer embryonic stem cell (ESC) (ntESC) lines including those from transgenic marmoset. We characterize these ntESCs at cytogenetic, molecular, and functional levels and compare them with fertilization-derived marmoset ESCs. Transcriptomic analyses further uncover subsets of developmentally regulated and ribosome-associated genes that are aberrantly expressed in ntESCs, providing insights into residual reprogramming defects and directions for further optimization of SCNT in primates.

RESULTS

Establishment of basic SCNT procedures in marmosets

We first established basic procedures of SCNT in marmosets based on the protocol reported in a previous paper (Sotomaru et al., 2009). Oocytes at the germinal vesicle stage were collected from ovaries of adult female marmosets following hormonal stimulation (Kurotaki and Sasaki, 2017) and underwent *in vitro* maturation to obtain matured metaphase II (MII) oocytes (Figure 1A). Chromosomes were removed from MII oocytes using micromanipulation in the presence of cytochalasin B (Figure 1B). DNA staining of the enucleated spindle within a small cytoplasm confirmed the effective enucleation (Figure 1C). We used adult fibroblasts derived from CAG-EGFP transgenic marmosets (here after referred to as GFP; Sasaki et al., 2009), as donor cells allowing for easy tracing of nuclear origin. These fibroblasts were cultured to confluency to enrich for cells in the G1/G0 phase and then fused with enucleated oocytes using inactivated hemagglutinating virus of Japan envelope (HVJ-E). The reconstructed oocytes were activated by ionomycin followed by 6-dimethylaminopurine (6-DMAP) treatment and cultured in a sequential embryo culture system (Figure 1A).

While intracytoplasmic sperm injection (ICSI) embryos developed into blastocysts at a rate of 35.4% in our institute (Takahashi et al., 2014), SCNT embryos derived from fibroblasts by the above method failed to progress beyond the 8-cell stage (Table 1). The SCNT embryos generated from cumulus cells of wild-type (WT) donor marmoset also arrested development before reaching the 8-cell stage (Table 1). This developmental arrest coincided with the timing of ZGA in marmosets (Boroviak et al., 2018), suggesting that insufficient reprogramming at this critical stage may underlie the failure of blastocyst formation, consistent with a previous report of marmosets (Sotomaru et al., 2009) and other species (Matoba and Zhang, 2018; Matoba et al., 2014). These results establish a baseline SCNT procedure in marmosets and highlight ZGA as a key bottleneck for developmental progression.

Kdm4d expression facilitates blastocyst formation in SCNT embryos

To overcome the developmental block observed at the 8-cell stage, we tested whether mRNA encoding mouse *Kdm4d* could improve SCNT embryo development in marmosets. We injected *Kdm4d* mRNA into SCNT embryos at 5–6 h after activation, and examined H3K9me3 levels at 5 h after mRNA

(C) The enucleated karyoplasm with spindle visualized by Hoechst 33342. White arrows indicate the karyoplasm containing spindle. Scale bar, 50 μm.

(D) Immunofluorescence images of the 1-cell stage ICSI- or SCNT-generated (clone) embryos stained with H3K9me3 antibody. Embryos were stained 10 h after activation (5 h after *Kdm4d* mRNA injection). Note that H3K9me3 was completely depleted upon mouse *Kdm4d* mRNA injection. Mat PN, maternal pronucleus; Pat PN, paternal pronucleus; PPN, pseudo-promnucleus. Scale bar, 10 μm.

(E) Morphology of the SCNT-generated blastocyst derived from donor fibroblasts of GFP-transgenic marmosets. The GFP signal confirms nuclear donor origin. Scale bar, 50 μm.

(F) Representative images of SCNT blastocysts stained with inner-cell-mass-specific marker, NANOG, and TE-specific marker, CDX2. Scale bar, 50 μm.

Table 1. Preimplantation development of marmoset SCNT embryos and derivation of ntESCs

Nuclear donor		Booster				Day 0	Day 1	Day 5–6		Day 7–10		ntESC establishment	
Genotype	Cell type	TSA	*Kdm4d*	G9ai	No. of oocyte donors	No. of reconstructed oocytes	No. of PN+	~7 cell	~8 cell	Blastocyst	Blastocyst rate (% per PN+)	No. of established ntESC lines	ntESC establishment rate (% per blast)
GFP	Fibroblast	+	−	−	2	6	6	2	0	0	0	–	–
		+	+	−	14	78	69	56	12	10	14.5	0	0.0
		+	−	+	4	13	13	11	2	0	0.0	0	0.0
		+	+	+	10	67	67	55	12	10	14.9	4	40.0
WT	Cumulus cell	+	−	−	12	34	30	13	0	0	0.0	–	–
		+	+	−	12	55	42	40	2	1	2.4	0	0.0
	Fibroblast	+	+	+	19	134	125	85	35	17	13.6	3	17.6

injection by immunostaining. In ICSI-generated embryos, H3K9me3 was specifically detected in the maternal pronucleus (PN) (Figure 1D), as reported previously (Ogonuki et al., 2018). Interestingly, in control SCNT embryos, H3K9me3 was strongly detected at the DAPI-dense regions preferentially localized near the nuclear membrane of pseudo-pronucleus (PPN), which is a PN-like structure formed in SCNT embryos at the 1-cell stage (Figure 1D). In contrast, mouse *Kdm4d* expression led to a global reduction of H3K9me3 signals in the PPN (Figure 1D), indicating effective demethylation of H3K9me3 by mouse KDM4D in the marmoset SCNT context.

Next, we assessed the developmental potential of the SCNT embryos. In the control, none of the embryos reached the blastocyst stage, as described above. In contrast, *Kdm4d* and TSA double-treated SCNT embryos developed to the blastocyst stage, at an efficiency of 14.5% per total PN stage embryos (Figure 1E; Table 1). Similar improvement was also observed using cumulus cells as donors, although the blastocyst rate (2.4%) was much lower than in fibroblasts (Table 1). Immunofluorescence staining of the resulting blastocysts revealed the presence of a NANOG-positive inner cell mass and CDX2-positive trophectoderm, confirming proper lineage segregation (Figure 1F). Furthermore, GFP fluorescence confirmed that the nuclear genome of these embryos originated from donor GFP-transgenic fibroblasts (Figures 1E and 1F). These results demonstrate that *Kdm4d* mRNA expression together with TSA treatment improves reprogramming and enables blastocyst formation from SCNT embryos in marmosets.

G9a inhibition further improves blastocyst quality and enables ntESC derivation

Since *Kdm4d* mRNA injection together with TSA treatment enabled the formation of morphologically normal blastocysts, we attempted to derive ESCs. Although the SCNT blastocysts initiated outgrowth, the expanded cells started to degenerate within a few days and failed to form colonies after passage (Figure 2D). This result suggested that residual epigenetic defects remain in SCNT embryos. Our preliminary data on embryo transfer of these SCNT embryos consistently failed to produce any live neonates (43 embryos were transferred to 27 recipient females in total).

We hypothesized that further epigenetic reprogramming might be necessary to improve embryo quality. Based on our recent findings in mice, we focused on a G9a/EHMT2 histone methyltransferase inhibitor (RK-701; G9ai) (Matoba et al., 2024), which reduces H3K9 methylation levels because of its highly specific inhibitory action on G9a (Nishigaya et al., 2023; Takase et al., 2023). We applied G9ai treatment to marmoset SCNT embryos in combination with *Kdm4d* mRNA and TSA treatment (Figure 2A).

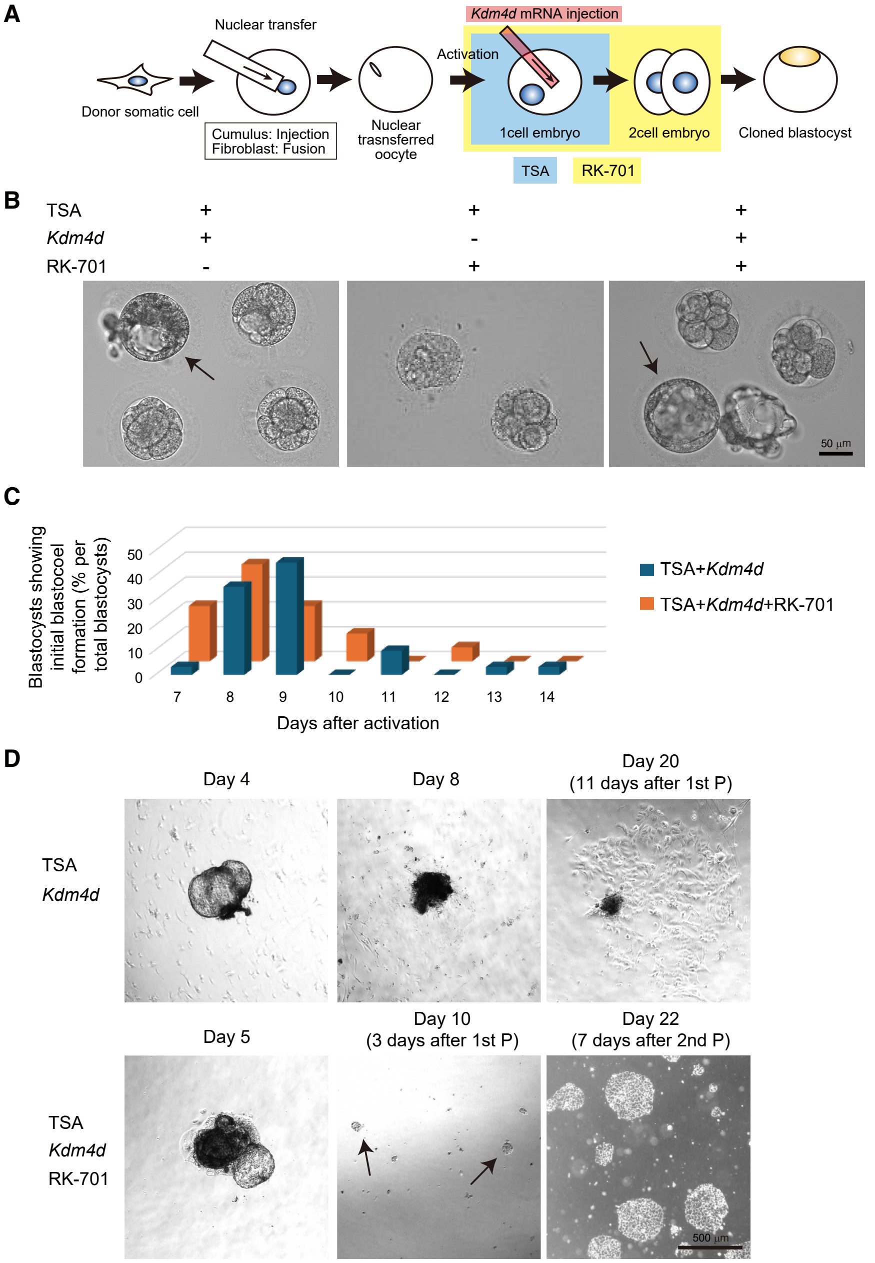

Figure 2. G9a inhibition improves the quality and yield of SCNT embryos

(A) Experimental scheme showing combination treatment of *Kdm4d* mRNA, TSA, and G9a inhibitor (RK-701).

(B) Representative morphology of SCNT blastocysts with or without G9a inhibitor treatment. Arrows indicate embryos that developed to the blastocyst stage. Scale bar, 50 μm. (C) Timing of initial blastocoel formation.

(D) Timeline of ntESC colony outgrowth from SCNT blastocysts. Note that SCNT blastocysts treated with TSA and *Kdm4d* failed to form colony after passage. G9a inhibition enhanced the quality of SCNT blastocysts enabling ntESC derivation. 1st P and 2nd P represent first passage and second passage, respectively. Arrows indicate small colonies that appeared after passage. Scale bar, 500 μm.

Although this combined triple treatment did not significantly improve the blastocyst formation rate of GFP-transgenic cloned embryos (14.9%; Table 1), the quality of the obtained blastocysts improved since the blastocoel formation speed, which is an indicator of developmental competency (Harada et al., 2020), was accelerated by this treatment (Figures 2B and 2C). When applied to WT fibroblast donors, the triple treatment protocol reproducibly yielded blastocysts at comparable efficiency (13.6%) to those from GFP-transgenic donors (Table 1).

We attempted to derive ntESCs from these blastocysts (Kishimoto et al., 2021). The SCNT blastocysts efficiently attached to the dish bottom and expanded (Figure 2D). We successfully derived 3 ntESC lines from WT fibroblast donors and 4 lines from GFP-transgenic donors (Tables 1 and S1). All ntESC lines maintained the flat colony morphology of primed ESC state (Figures 3A and 3B) and sustained proliferation over multiple passages (more than 17 passages over a period of two months) (Figure S1A). These lines included both male XY and female XX chromosomal donor origins (Figures 3C and 3D; Table S1). Karyotyping revealed normal chromosomal content in all 7 lines at passage (P) 6, with one exception of line #54 showing a translocation abnormality [t(7p;15q)] after 11 passages

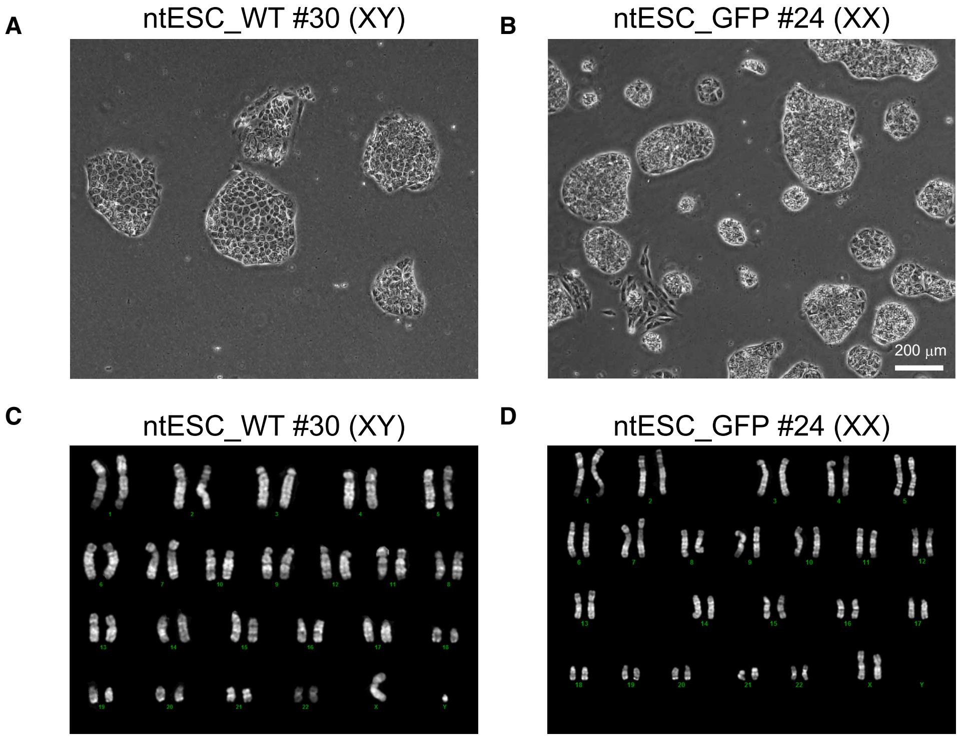

E

		2463-TH		CJ060		CJ077		CJ081		CJ103		CJ187		CJ003		CJ083		CJ091		CJ146		CjX		CjY
		Allele 1	Allele 2	Allele 1	Allele 2	Allele 1	Allele 2	Allele 1	Allele 2	Allele 1	Allele 2	Allele 1	Allele 2	Allele 1	Allele 2	Allele 1	Allele 2	Allele 1	Allele 2	Allele 1	Allele 2	Allele 1	Allele 2	Allele 1
WT (#30)	Donor	109	109	135	149	207	209	170	186	113	119	201	207	88	88	123	125	135	147	132	134	204		208
	ntESC	109	109	135	149	207	209	170	186	113	119	201	207	88	88	123	125	135	147	132	134	204		208
	Oocyte	109	109	135	147	205	209	168	184	109	119	201	207	84	96	123	123	139	139	134	146	190	204	
GFP (#24)	Donor	109	109	135	147	197	205	160	186	89	119	201	201	88	94	121	125	139	139	134	148	190	200	
	ntESC	109	109	135	147	197	205	160	186	89	119	201	201	88	94	121	125	139	139	134	148	190	200	
	Oocyte	109	109	135	135	205	211	186	186	111	127	201	207	84	98	123	123	139	145	134	141	190	208	

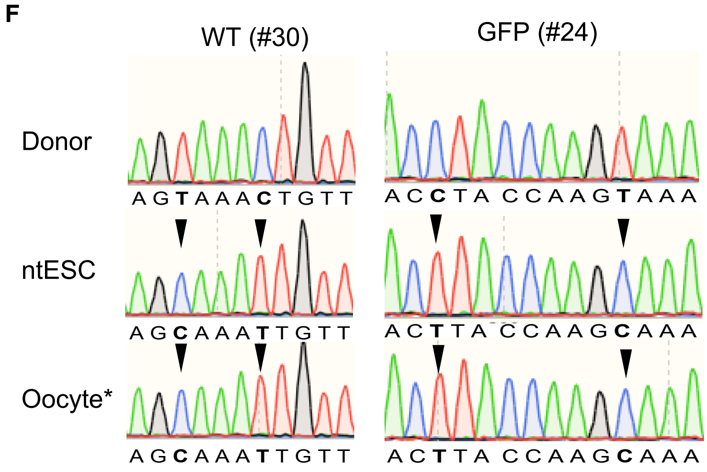

Figure 3. Derivation and genetic validation of ntESC lines

(A and B) Morphology of ntESCs derived from (A) wild-type (#30, XY) and (B) GFP-transgenic (#24, XX) fibroblasts. Scale bar, 200 μm.
(C and D) Karyotype of ntESCs derived from (C) wild-type (#30) and (D) GFP-transgenic (#24, XX) fibroblasts.

(legend continued on next page)

(Table S1). Together, these results demonstrate that G9a inhibition enhances SCNT embryo quality and enables the successful derivation of stable ntESC lines in the common marmoset.

Confirmation of nuclear and mitochondrial origins of ntESCs

To determine the nuclear genotype, we performed haplotype analysis by microsatellite markers. In ntESC WT line #30, the nuclear genotype of all 12 microsatellite markers matched that of the somatic donor fibroblasts but was distinct from the oocyte donor (Figure 3E). Similarly, all microsatellite markers of ntESC GFP line #24 matched donor somatic cells but not with oocytes (Figure 3E). These results confirmed successful replacement of the oocyte genome with the somatic donor nucleus. We also sequenced the mitochondrial D-loop region to assess cytoplasmic inheritance from recipient oocytes. The D-loop sequence of each ntESC line matched that of the recipient oocyte but was distinct from the somatic cell donor (Figure 3F), indicating cytoplasmic inheritance from the recipient oocyte. All seven ntESC lines analyzed showed consistent results, confirming that they truly were SCNT-derived ntESCs with somatic cell-derived nuclear genomes and oocyte-derived mitochondria. These findings confirm that the ntESC lines were authentically derived from SCNT embryos.

Characterization of ntESCs: Pluripotency and differentiation potential

Next, we assessed the pluripotency of ntESC lines using immunofluorescence and transcriptomic analyses. Immunostaining revealed uniform expression of key pluripotency markers, including NANOG, POU5F1, and SOX2 (Figure 4A), comparable to fertilization-derived ESCs (Kishimoto et al., 2021). To further examine gene expression profiles, we performed RNA sequencing (RNA-seq) on ntESCs, donor fibroblasts, and fertilization-derived control ESCs (*in vitro*-fertilized [IVF] ESCs and naturally fertilized [NAT] ESCs). Principal-component analysis (PCA) showed that ntESCs were transcriptionally distinct from donor fibroblasts and clustered closely with control ESCs (Figure 4B). While *DCN*, which is highly expressed in the donor fibroblasts, was completely silenced in the ntESCs, pluripotency-related genes such as *POU5F1* and *NANOG* were expressed in ntESCs at similar levels with control ESCs (Figure 4C).

To evaluate the differentiation potential of ntESCs, we induced embryoid body (EB) formation *in vitro*. Immunostaining of ntESC-derived EBs (ntEBs) with differentiation markers indicated that these ntESCs could differentiate into all three germ layers (Figures S1B and S1C). Comparative transcriptome analysis of ntESCs and their derivative ntEBs revealed consistent downregulation of pluripotency markers and upregulation of markers for all three germ layers, including the ectoderm, endoderm, and mesoderm in control EBs (Figure 5A). The general trend was similar in ntEBs of WT and GFP. However, we noted that pluripotent marker genes were not clearly downregulated upon differentiation in the ntESC GFP #24 line. Moreover, many of the differentiated lineage markers were unevenly expressed in ntEB, while these showed more consistent expression in control EB (Figure 5A). We also noticed that some of the differentiation markers were aberrantly expressed in undifferentiated ntESCs when compared to control ESCs: *PAX6* in ntESC GFP #24 and #54, *CFTR* in ntESC GFP #54, *GATA2* in ntESC WT #30, and ntESC GFP #50 (Figure 5A). In contrast, some ntESC lines showed preferential differentiation to a specific lineage; ectoderm differentiation was robust in ntESC #61 but less efficient in ntESC GFP #24. These results suggest lineage-biased or unstable pluripotent states in ntESCs.

We also performed teratoma formation assays by injecting ntESCs into the kidney capsule of immunodeficient mice. Teratoma was efficiently formed at 4–7 weeks after transplantation of ntESCs (Figure 5B). Histological analysis confirmed the presence of representative tissues of all three germ layers in the ntESC-derived teratoma (Figure 5C). Notably, endodermal and mesodermal tissues were well differentiated, whereas ectodermal components showed immature or degenerated features in ntESC GFP #24 line and even in control ESC #11 (Figure 5C). These results demonstrate that ntESCs derived from SCNT blastocysts possess pluripotent characteristics and differentiation capacity comparable to conventional ESCs, albeit with a line-dependent lineage bias in differentiation.

Identification of reprogramming-resistant genes for further improvement

Despite successful derivation of ntESCs, embryo transfer of the SCNT blastocysts generated by our optimized method failed to produce any neonates (12 embryos were transferred to 9 recipient females in total). Previous studies in mice, monkeys, and humans have reported that *Kdm4d*- or G9ai-boosted SCNT-generated embryos and ntESCs

(E) Microsatellite markers analyzed by PCR. Each number indicates the size (base pairs) of PCR product. Note that all microsatellite marker sizes of ntESCs are identical to those of donor cells but not with recipient oocytes.
(F) Mitochondrial D-loop sequencing of donor cells, ntESCs, and recipient oocytes. Shown are two positions in the hypervariable D-loop with single-nucleotide polymorphism differences between donor cells and recipient oocytes. See also Table S1.

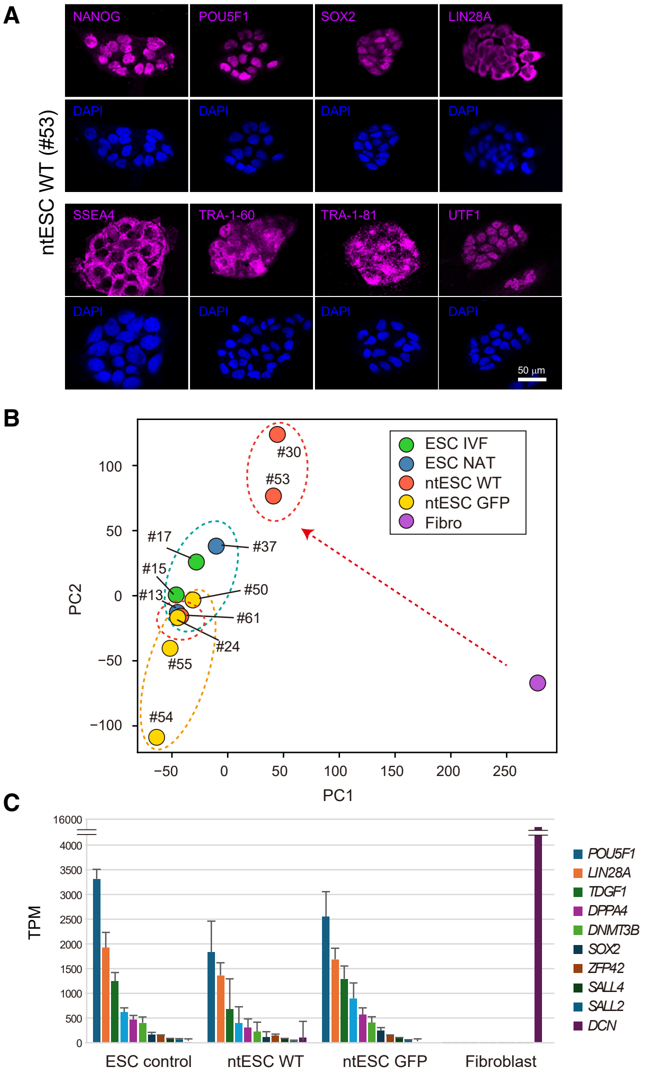

Figure 4. Pluripotency marker expression and transcriptomic profiling of ntESCs

(A) Immunofluorescence staining of ntESC WT for pluripotent marker proteins, NANOG, POU5F1, SOX2, LIN28A, SSEA4, TRA-1-60, TRA-1-81, and UTF1. Scale bar, 50 μm.

(B) Principal-component analysis of transcriptome of ESC, ntESC, and donor fibroblasts. Note that ntESCs show close similarity with control ESCs, but not with fibroblasts (Fibro). Red dashed arrow represents reprogramming of donor WT fibroblasts to ntESC WT lines.

(C) Expression levels of pluripotency-related genes and fibroblast marker gene, DCN, in ESC, ntESC, and donor fibroblasts. Transcripts per million (TPM) values are shown. Error bars represent standard deviation. See also Table S2.

harbor additional epigenetic abnormalities (Chung et al., 2015; Liao et al., 2024; Matoba and Zhang, 2018).

To explore this possibility in marmosets, we analyzed the transcriptomes of ntESCs and ntEBs to identify genes that may have escaped full reprogramming through SCNT and ntESC derivation processes. Differential gene expression analysis between ntESCs (WT and GFP) and fertilization-derived control ESCs revealed 752 upregulated and 350 downregulated genes in ntESCs (fold change >2) (Figure 6A; Table S2). Gene Ontology (GO) analysis of the upregulated 752 genes indicated significant enrichment for pathways related to embryonic development, such as "anterior/posterior pattern specification" and "embryonic skeletal system morphogenesis" (Figure 6B). Interestingly, these terms mostly consisted of a group of homeobox (*HOX*) genes including *HOX4A*, *HOXC5*, and *HOXC8*. Such upregulation of *HOX* genes was observed in a line-dependent manner, as only ntESC WT #30, ntESC WT #53, and ntESC GFP #50 showed widespread upregulation of these genes (Figure 6C). A similar line-dependent upregulation of *HOX* genes was observed in ntEBs (Figure S2; Table S3). We also found that genes termed "translation" were significantly enriched in the upregulated genes (Figure 6B). While genes in this category were mostly uncharacterized, they were all related to ribosomal protein variants such as *40S ribosomal protein S15-like* (*RPS15-like*: *LOC108589403*) and *60S ribosomal protein L21-like* (*RPL21-like*: *LOC118153695*) (Figure 6D).

In turn, GO analysis of the downregulated 350 genes indicated significant enrichment of immune response. These mostly consisted of immune-regulatory cytokines such as *CXCL1*, *CXCL2*, and *CXCL8*. Interestingly, despite widespread upregulation of ribosomal proteins as described above, some other ribosomal protein variants such as *RPL3-like* (*LOC103793262*) and *RPS2-like* (*LOC108592091* and *LOC100394437*) were downregulated in ntESCs. These results suggest that although the global gene expression pattern of ntESCs resembles that of IVF-derived ESCs (Figure 4B), a subset of genes remains incompletely reprogrammed and may underlie the reduced developmental potential of SCNT embryos.

DISCUSSION

In this study, we established ntESC lines from common marmoset SCNT embryos using a combinatorial epigenetic enhancement protocol that included TSA treatment, *Kdm4d* mRNA injection, and G9a/EHMT2 inhibition. This strategy enabled the derivation of blastocysts and stable ntESC lines from both WT and GFP-transgenic donor cells, representing a significant breakthrough in overcoming the developmental arrest that has previously limited SCNT in marmosets. The ntESC lines displayed typical primed marmoset ESC morphology, expressed core pluripotency markers, and propagated over the long term. Karyotypes were largely stable across early passages, with only one line showing a structural rearrangement at a later passage.

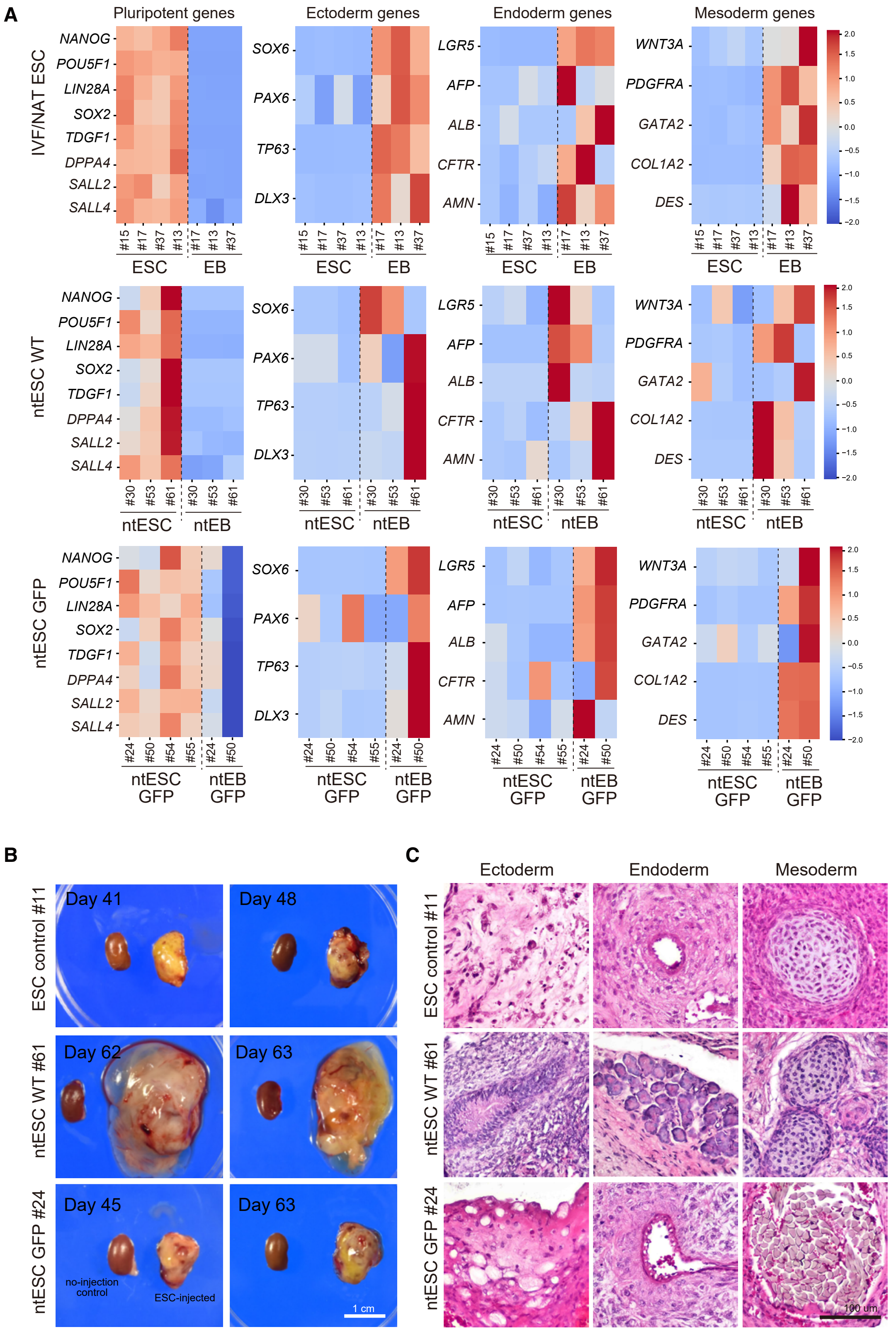

(legend on next page)

Nuclear microsatellite genotyping and mitochondrial D-loop sequencing verified that these cell lines act as bona fide SCNT-derived cell lines. Together, these data demonstrate that our optimized protocol reliably captures the donor nuclear genome in a pluripotent state.

How TSA, *Kdm4d*, and G9a inhibition act synergistically to improve reprogramming remains a key mechanistic question. Each of these agents targets distinct aspects of the repressive H3K9 chromatin landscape. TSA promotes global histone acetylation and chromatin accessibility, facilitating transcriptional reactivation after nuclear transfer (Kishigami et al., 2006). *Kdm4d* actively removes this repressive mark at key loci, including ZGA regions, thereby enhancing developmental gene expression (Matoba et al., 2014). G9a inhibition further reduces H3K9me2/3 levels by blocking the activity of a major H3K9 methyltransferase (Matoba et al., 2024). The combinatorial use of these agents may thus exert complementary effects. This multi-pronged approach, targeting H3K9 methylation, could allow more complete resetting of epigenetic memory and support more faithful reprogramming of somatic nuclei toward pluripotency.

Transcriptomic comparisons revealed notable differences between ntESCs and control ESCs, with some patterns being conserved across lines, while others appeared more line specific. Among the more variable changes, some ntESC lines exhibited precocious and clustered upregulation of HOX genes (Figure 6C), which are normally repressed in the pluripotent state and activated later during differentiation (Hubert and Wellik, 2023). Their aberrant activation in specific ntESC lines likely reflects incomplete silencing of somatic gene expression programs that were highly expressed in donor fibroblasts. Since these HOX genes are physically clustered in the genome, the phenomenon may also be influenced by regional epigenetic resistance to reprogramming, such as incomplete resetting of topologically associating domains (Lonfat and Duboule, 2015). Similarly, a subset of CXCL genes was downregulated in certain ntESC lines (Figure 6H), and these also tended to form genomic clusters. The presence of these expression clusters points to the importance of further studies on chromatin domain remodeling during reprogramming.

Interestingly, ribosome-associated genes showed a more consistent trend across ntESC lines (Figures 6D and 6G). Both up- and downregulation of genes encoding 40S and 60S ribosomal subunits were detected, suggesting widespread perturbation of the translational machinery. Notably, alterations in ribosomal gene expression have been implicated in a class of disorders known as ribosomopathies (Kang et al., 2021). Subtle imbalances in ribosome biogenesis and function can also affect stem cell lineage commitment and genome integrity via the p53 pathway (Zhou et al., 2015). The consistent dysregulation of ribosomal protein genes may reflect a common vulnerability of reprogrammed cells to defective ribosome homeostasis. Further investigation is warranted to determine whether ribosome-associated abnormalities contribute to the functional limitations of ntESCs in primates.

In summary, this study demonstrated that an optimized combination of epigenetic modifiers, TSA, *Kdm4d*, and G9ai, all of which target H3K9 methylation, enabled efficient production of blastocysts through SCNT and led to the successful derivation of ntESCs in the common marmoset. Beyond H3K9me3, persistent H3K4me3 (Liu et al., 2016), aberrant DNA methylation (Gao et al., 2018; Matoba et al., 2018), and failures in non-canonical imprinting governed by H3K27me3 also impair developmental potential in mice (Inoue et al., 2020; Matoba et al., 2018, 2022; Wang et al., 2020). Although these mechanisms remain to be investigated in the marmoset, overcoming these targets may enhance cloning efficiency and pluripotent cell quality in marmosets. More importantly, improving oocyte quality by using *in vivo* matured MII oocytes may further enhance the overall efficiency of embryonic development following SCNT. Similarly, finding the most suitable donor cell type for SCNT may help increase efficiency of reprogramming, as cumulus cells obtained from *in vitro* matured oocytes showed unexpectedly low efficiency. Together, our findings lay the foundations for improving nuclear reprogramming in the marmoset and suggest that broader epigenetic editing strategies may be required to fully reset somatic memory and unlock the full developmental potential of SCNT-derived embryos and ESCs in primates.

METHODS

Animals

All animal experiments were approved by the Institutional Animal Care and Use Committee of the Central Institute

Figure 5. *In vitro* and *in vivo* differentiation potential of ntESCs
(A) Heatmap showing the average expression levels of pluripotent genes and three germ-layer genes in ntESCs and their derivative ntEBs.
(B) Representative images of teratoma formed under kidney capsules. Scale bar, 1cm.
(C) Histology of teratoma. Sections were stained with H&E. Shown are representative tissues of the three germ layers, ectoderm, endoderm, and mesoderm, formed in teratoma. Note that the ectoderm-like tissues in ESC control #11 and ntESC GFP #24 were immature/degenerated neural tissues and epithelial tissues, respectively, while ntESC WT #61 efficiently formed all three germ layers. Scale bar, 100 μm. See also Figure S1 and Tables S2 and S3.

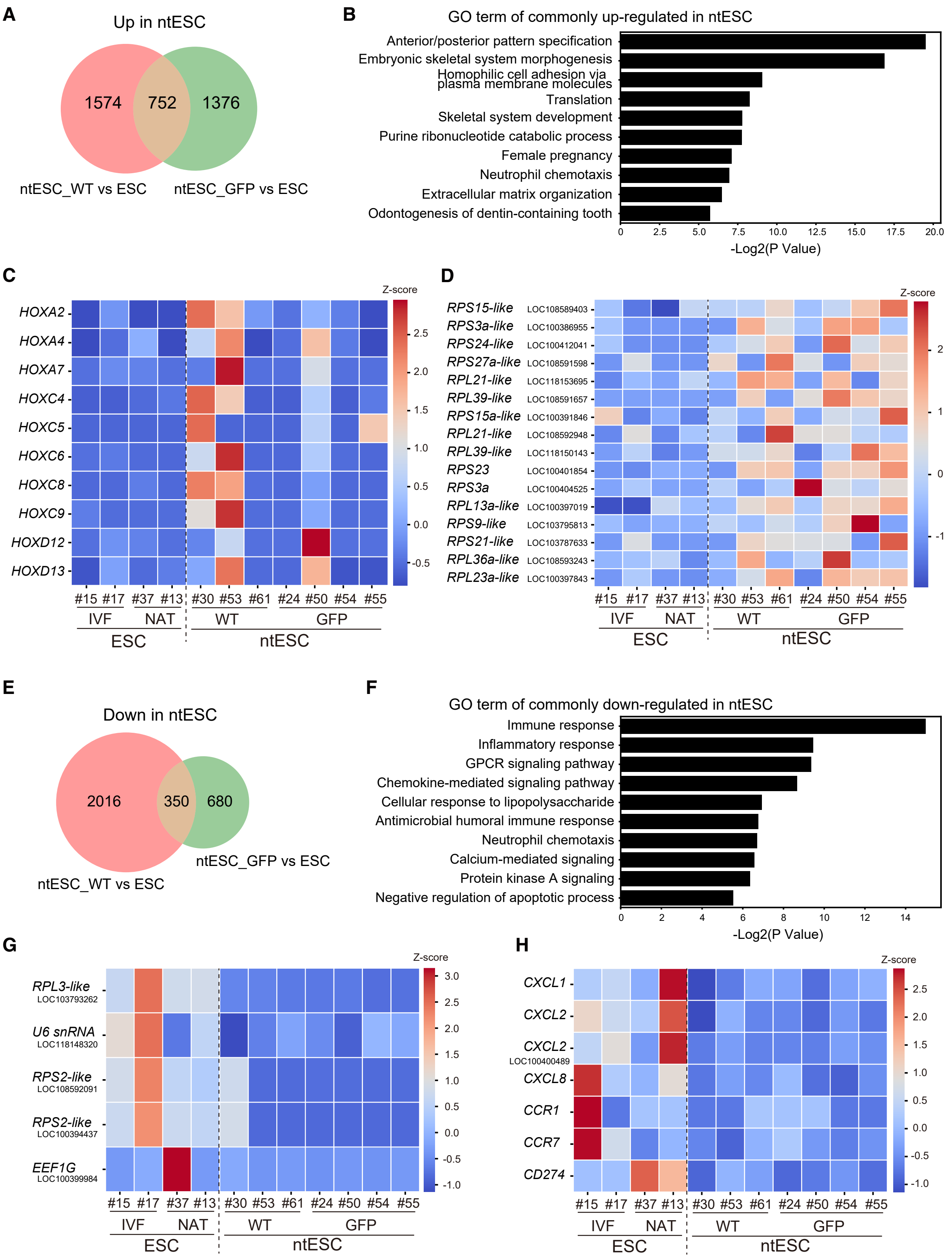

(legend on next page)

for Medicine and Life Science in Japan (approval numbers: 2020–2023 20049A, 2024–2025 AIA240014, and AIA240127) and were conducted in accordance with institutional and national guidelines. Adult female common marmosets were obtained from CLEA Japan, Inc., National Center of Neurology and Psychiatry, and Primate Research Institute of Kyoto University. NOG (NOD/Shi-scid, IL-2Rγ null) mice were purchased from CLEA Japan, Inc.

Somatic cell nuclear transfer

MII oocytes were enucleated in HEPES-buffered CZB medium containing cytochalasin B. Cumulus cells were pipetted though a thin glass needle to isolate nuclei, which were then directly injected into the enucleated oocyte cytoplasm using piezo-assisted micromanipulation, without requiring cell fusion. For fibroblasts, membrane-intact cells were inserted to the perivitelline space of the enucleated oocytes with HVJ-E (Ishihara Sangyo, Japan) to induce donor cell-ooplasm fusion. After reconstitution, oocytes were rested in porcine oocyte medium (IFP1010P, Research Institute for the Functional Peptides, Japan) for 1 h and then activated by 5-min exposure to ionomycin, followed by 6-h culture in medium containing 6-DMAP and TSA. TSA treatment was continued for an additional 3 h (8 h total). *Kdm4d* mRNA was injected at a concentration of 1,500 ng/μL (∼10–20 pL per embryo) 5–6 h post-activation (Matoba et al., 2014; 2018). Embryos were then cultured in Sequential Cleav medium (83040010A, CooperSurgical, USA) until 8-cell stage, followed by transfer to Sequential Blast medium (83050010A, CooperSurgical). In some experiments, 1 μM RK-701 (G9a/EHMT2 inhibitor; Matoba et al., 2024) was added at the time of activation and maintained for 24 h.

Derivation and culture of ntESC lines

ntESCs were established by the feeder-free protocol (Kishimoto et al., 2021). In short, SCNT blastocysts (day 8–12) were treated with acidified Tyrode's solution (10605000, CooperSurgical) to remove the zona pellucida. Embryos were plated onto iMatrix-551 silk (387-1013, Fujifilm Wako Pure Chemical Corporation, Japan)-coated plates (Nippi, Japan; 0.5 μg/cm^2) and cultured in conditioned medium for ESC (CMESC) supplemented with 4 ng/mL human recombinant basic fibroblast growth factor (bFGF) (RCHEOT002, REPROCELL, Japan). CMESC was prepared by culturing irradiated MEF feeder cells in Primate ES Cell Medium (RCHEMD001, REPROCELL) for 24 h. Outgrowths were observed within 4–10 days. When the cell colonies expanded, they were dissociated using Accutase (12679-54, Nacalai Tesque, Japan) and passaged onto new iMatrix-coated plates. After every passage, Y-27632 (#72308, Stemcell Technologies Inc., Canada) was added to the culture medium at 10 μM for 1 day to support the survival of dissociated ESCs. Media were replaced every 1–2 days.

Embryoid body formation and teratoma assay

To evaluate *in vitro* differentiation potential, ESCs or ntESCs were dissociated using Accutase, and 1–3 $\times$ 10^6 cells were cultured in non-adherent dishes with the Primate ESC Medium containing 5% KnockOut Serum Replacement (KSR, 10828010, Thermo Fisher Scientific, USA). Y-27632 (50 μM) was added on day 1. EBs were cultured for 30 days, and samples were harvested for immunostaining and RNA-seq.

For *in vivo* differentiation, teratoma assays were performed by injecting 1 $\times$ 10^6 ESCs/ntESCs into the kidney capsule of 8-week-old immunodeficient NOG mice. After 6–10 weeks, teratomas were excised, fixed in 4% paraformaldehyde (09154-85, Nacalai Tesque), embedded in paraffin, sectioned, and stained with H&E for histological analysis.

Karyotype analysis

Karyotype analysis was performed on all ntESC lines using a modified Q-banding method (Sasaki et al., 2005; Sugawara et al., 2006). Cells in logarithmic growth phase were treated with 100–200 ng/mL colcemid (15212012, Thermo Fisher Scientific) for 90–180 min at 37°C in 5% CO_2. Then the cells were harvested using Accutase and resuspended in 0.075 M KCl (10575090, Thermo Fisher Scientific) for 20 min at room temperature for hypotonic treatment. After fixation in methanol:acetic acid (3:1), the cells were dropped onto glass slides and air-dried overnight.

For chromosome counting, slides were stained with 5% Giemsa solution for 10 min, and metaphase spreads were

Figure 6. Transcriptome-wide identification of abnormally expressed genes in ntESCs
(A) Venn diagram showing the number of upregulated genes in ntESC WT and ntESC GFP compared to control ESC.
(B) GO term enriched in commonly upregulated genes in ntESCs.
(C) Heatmap showing the expression levels of upregulated HOX-related genes.
(D) Heatmap showing the expression levels of upregulated ribosomal protein-related genes.
(E) Venn diagram showing the number of downregulated genes in ntESC WT and ntESC GFP compared to control ESC.
(F) GO term enriched in commonly downregulated genes in ntESCs.
(G) Heatmap showing the expression levels of downregulated ribosomal protein/translation-related genes.
(H) Heatmap showing the expression levels of downregulated immune response-related genes. See also Figure S2 and Table S2.

examined under a microscope. At least 28 metaphases were analyzed per line to determine the modal chromosome number. Structural abnormalities were detected using quinacrine mustard (50 μg/mL) and Hoechst 33258 (23491-45-4, DOJINDO, Japan) staining, followed by fluorescence microscopy (DM6000B, Leica, Germany) and imaging with the Leica CytoVision system (Leica). Five or more metaphases were examined for each line.

DNA extraction and genotyping

Genomic DNA was extracted from donor fibroblasts, established ntESCs, and recipient oocyte donors (hair roots). Samples were incubated overnight at 55°C in lysis buffer (100 mM Tris-HCl pH 7.5, 20 mM EDTA, 165 mM NaCl, 1% SDS, and 70 μg/mL proteinase K). DNA was extracted with phenol:chloroform:isoamyl alcohol and precipitated with ethanol.

Microsatellite genotyping was performed using primers specific to 12 polymorphic microsatellite markers. The analysis method for microsatellite markers located on 10 autosomes has been described previously (Takahashi et al., 2014). For the sex chromosomes, the primers were newly designed in the present study (Table S4). PCR products were analyzed using a SeqStudio8 Genetic Analyzer (Thermo Fisher Scientific) with GS500 LIZ size standard, and allele sizes were determined using GeneMapper 4.0 software (Thermo Fisher Scientific).

Mitochondrial DNA analysis

The mitochondrial DNA (mtDNA) of ntESCs, donor fibroblasts, and oocyte donors were analyzed by sequencing the hypervariable D-loop region. mtDNA was amplified by PCR using the following primers:

- CJ-D-loop-F1: 5′-GGAGAGAATATTTAATTCCACC-3′.
- CJ-D-loop-R1: 5′-GTTTGAGGTATGCGAGGAGTAACGG-3′.

(Reference: Accession AB525908, unpublished, Takabayashi and Katoh). PCR was performed using KOD One (KMM-101, TOYOBO, Japan) with a two-step protocol (98°C for 10 s, 60°C for 20 s, 30 cycles). PCR products were separated by 1.5% agarose gel electrophoresis, purified, and sequenced directly using the same primers.

RNA-seq and transcriptome analysis

RNA-seq was performed on IVF/NAT ESCs between passages P7 and P21 and on ntESCs between passages P5 and P10. Total RNA was extracted from ESCs/ntESCs, fibroblasts, and EBs/ntEBs using RNeasy Mini kit (74104, Qiagen GmbH, Germany). Library preparation and sequencing were performed using a TruSeq stranded mRNA library prep kit (RS-122-2101, Illumina, USA) and sequenced on NovaSeq 6000 (Illumina) or NovaSeq X Plus (Illumina) to generate 100-bp paired-end reads.

Adaptor and low-quality sequences were removed using fastp (version 0.20.1) with the "-l 20 -q 20" options (Chen et al., 2018). The remaining reads were mapped to marmoset genome (calJac4) using STAR (version 2.7.8a) with the "–outFilterMultimapNmax 1 –outFilterMismatchNmax 1" option (Dobin et al., 2013). Uniquely mapped read counts for each gene were calculated using featureCounts (subread version 2.0.6) (Liao et al., 2019), and then transcripts per million in each gene were calculated using Python. Sex chromosomes were excluded in this analysis because the proportion of male and female cells differs between conditions. Bar plots, PCA plots, Venn diagrams and heatmaps were generated using Python. Genes exhibiting at least a 2-fold increase or decrease of expression in ntESC WT or ntESC GFP compared to IVF/NAT ESC were defined as differentially expressed genes (DEGs). GO analysis was performed using DAVID (Huang et al., 2009).

RESOURCE AVAILABILITY

Lead contact

Requests for further information and resources should be directed to and will be fulfilled by the lead contact, Shogo Matoba (shogo.matoba@riken.jp).

Materials availability

All ntESCs generated in this study are available from the lead contact with a completed materials transfer agreement.

Data and code availability

The accession number for the RNA-seq data reported in this paper is GEO: GSE305965.

ACKNOWLEDGMENTS

We thank Dr. Keiko Kishimoto for providing marmoset ESCs, Dr. Hiroshi Suemizu for helpful discussion, and Ayano Tsukahara for technical assistance. This study was supported by Japan Agency for Medical Research and Development (AMED) under grant number 21dm0207118h0001 (S.M.), 19dm0207065h0001 (E.S.) (Brain Mapping by Integrated Neurotechnologies for Disease Studies [Brain/MINDS]), and JSPS KAKENHI grant numbers JP25K02201 (S.M.), JP25H01356 (S.M.), JP19H05759 (E.S.), and JP19H05758 (A.O.).

AUTHOR CONTRIBUTIONS

S.M., Y.K., E.S., and A.O. conceived the project and designed the experiments. S.M. and Y.K. performed most of the experiments. S.F. and S.M. analyzed the RNA-sequencing data. Y.Y. and N.O. helped in the SCNT experiments. H.S. and M.Y. performed nuclear and mitochondrial DNA analysis. N.Y., T.H., and Y.H. performed teratoma formation assay. S.M. and Y.K. wrote the manuscript.

DECLARATION OF INTERESTS

The authors declare no competing interests.

SUPPLEMENTAL INFORMATION

Supplemental information can be found online at https://doi.org/10.1016/j.stemcr.2025.102710.

Received: August 22, 2025
Revised: October 15, 2025
Accepted: October 15, 2025
Published: November 13, 2025

REFERENCES

Abe, Y., Nakao, H., Goto, M., Tamano, M., Koebis, M., Nakao, K., and Aiba, A. (2021). Efficient marmoset genome engineering by autologous embryo transfer and CRISPR/Cas9 technology. Sci. Rep. *11*, 20234. https://doi.org/10.1038/s41598-021-99656-4.

Blaschke, K., Ebata, K.T., Karimi, M.M., Zepeda-Martínez, J.A., Goyal, P., Mahapatra, S., Tam, A., Laird, D.J., Hirst, M., Rao, A., et al. (2013). Vitamin C induces Tet-dependent DNA demethylation and a blastocyst-like state in ES cells. Nature *500*, 222–226. https://doi.org/10.1038/nature12362.

Boroviak, T., Stirparo, G.G., Dietmann, S., Hernando-Herraez, I., Mohammed, H., Reik, W., Smith, A., Sasaki, E., Nichols, J., and Bertone, P. (2018). Single cell transcriptome analysis of human, marmoset and mouse embryos reveals common and divergent features of preimplantation development. Development *145*, dev167833. https://doi.org/10.1242/dev.167833.

Chen, S., Zhou, Y., Chen, Y., and Gu, J. (2018). fastp: an ultra-fast all-in-one FASTQ preprocessor. Bioinformatics *34*, i884–i890. https://doi.org/10.1093/bioinformatics/bty560.

Chung, Y.G., Matoba, S., Liu, Y., Eum, J.H., Lu, F., Jiang, W., Lee, J. E., Sepilian, V., Cha, K.Y., Lee, D.R., et al. (2015). Histone demethylase expression enhances human somatic cell nuclear transfer efficiency and promotes derivation of pluripotent stem cells. Cell Stem Cell *17*, 758–766. https://doi.org/10.1016/j.stem.2015.10.001.

Dobin, A., Davis, C.A., Schlesinger, F., Drenkow, J., Zaleski, C., Jha, S., Batut, P., Chaisson, M., and Gingeras, T.R. (2013). STAR: ultrafast universal RNA-seq aligner. Bioinformatics *29*, 15–21. https://doi.org/10.1093/bioinformatics/bts635.

Fang, X., Tanga, B.M., Bang, S., Seong, G., Saadeldin, I.M., Qamar, A.Y., Shim, J., Choi, K., Lee, S., and Cho, J. (2022). Vitamin C enhances porcine cloned embryo development and improves the derivation of embryonic stem-like cells. Reprod. Biol. *22*, 100632. https://doi.org/10.1016/j.repbio.2022.100632.

Gao, R., Wang, C., Gao, Y., Xiu, W., Chen, J., Kou, X., Zhao, Y., Liao, Y., Bai, D., Qiao, Z., et al. (2018). Inhibition of aberrant DNA remethylation improves post-implantation development of somatic cell nuclear transfer embryos. Cell Stem Cell *23*, 426–435.e5. https://doi.org/10.1016/j.stem.2018.07.017.

Harada, Y., Maeda, T., Fukunaga, E., Shiba, R., Okano, S., Kinutani, M., and Horiuchi, T. (2020). Selection of high-quality and viable blastocysts based on timing of morula compaction and blastocyst formation. Reprod. Med. Biol. *19*, 58–64. https://doi.org/10.1002/rmb2.12302.

Heide, M., Haffner, C., Murayama, A., Kurotaki, Y., Shinohara, H., Okano, H., Sasaki, E., and Huttner, W.B. (2020). Human-specific ARHGAP11B increases size and folding of primate neocortex in the fetal marmoset. Science *369*, 546–550. https://doi.org/10.1126/science.abb2401.

Himaki, T., Mori, H., Mizobe, Y., Miyoshi, K., Sato, M., Takao, S., and Yoshida, M. (2010). Latrunculin A Dramatically Improves the Developmental Capacity of Nuclear Transfer Embryos Derived from Gene-Modified Clawn Miniature Pig Cells. Cell. Reprogram. *12*, 127–131. https://doi.org/10.1089/cell.2009.0066.

Huang, D.W., Sherman, B.T., and Lempicki, R.A. (2009). Systematic and integrative analysis of large gene lists using DAVID bioinformatics resources. Nat. Protoc. *4*, 44–57. https://doi.org/10.1038/nprot.2008.211.

Hubert, K.A., and Wellik, D.M. (2023). Hox genes in development and beyond. Development *150*, dev192476. https://doi.org/10.1242/dev.192476.

Inoue, K., Ogonuki, N., Kamimura, S., Inoue, H., Matoba, S., Hirose, M., Honda, A., Miura, K., Hada, M., Hasegawa, A., et al. (2020). Loss of H3K27me3 imprinting in the Sfmbt2 miRNA cluster causes enlargement of cloned mouse placentas. Nat. Commun. *11*, 2150. https://doi.org/10.1038/s41467-020-16044-8.

Inoue, T., Yurimoto, T., Seki, F., Sato, K., and Sasaki, E. (2023). The common marmoset in biomedical research: experimental disease models and veterinary management. Exp. Anim. *72*, 140–150. https://doi.org/10.1538/expanim.22-0107.

Kang, J., Brajanovski, N., Chan, K.T., Xuan, J., Pearson, R.B., and Sanij, E. (2021). Ribosomal proteins and human diseases: molecular mechanisms and targeted therapy. Signal Transduct. Target. Ther. *6*, 323. https://doi.org/10.1038/s41392-021-00728-8.

Kishigami, S., Mizutani, E., Ohta, H., Hikichi, T., Thuan, N.V., Wakayama, S., Bui, H.-T., and Wakayama, T. (2006). Significant improvement of mouse cloning technique by treatment with trichostatin A after somatic nuclear transfer. Biochem. Biophys. Res. Commun. *340*, 183–189. https://doi.org/10.1016/j.bbrc.2005.11.164.

Kishimoto, K., Shimada, A., Shinohara, H., Takahashi, T., Yamada, Y., Higuchi, Y., Yoneda, N., Suemizu, H., Kawai, K., Kurotaki, Y., et al. (2021). Establishment of novel common marmoset embryonic stem cell lines under various conditions. Stem Cell Res. *53*, 102252. https://doi.org/10.1016/j.scr.2021.102252.

Kurotaki, Y., and Sasaki, E. (2017). Practical reproductive techniques for the common marmoset. J. Mamm. Ova Res. *34*, 3–12. https://doi.org/10.1274/032.034.0103.

Liao, Y., Smyth, G.K., and Shi, W. (2019). The R package Rsubread is easier, faster, cheaper and better for alignment and quantification of RNA sequencing reads. Nucleic Acids Res. *47*, e47. https://doi.org/10.1093/nar/gkz114.

Liao, Z., Zhang, J., Sun, S., Li, Y., Xu, Y., Li, C., Cao, J., Nie, Y., Niu, Z., Liu, J., et al. (2024). Reprogramming mechanism dissection and trophoblast replacement application in monkey somatic cell nuclear transfer. Nat. Commun. *15*, 5. https://doi.org/10.1038/s41467-023-43985-7.

Liu, W., Liu, X., Wang, C., Gao, Y., Gao, R., Kou, X., Zhao, Y., Li, J., Wu, Y., Xiu, W., et al. (2016). Identification of key factors conquering developmental arrest of somatic cell cloned embryos by combining embryo biopsy and single-cell sequencing. Cell Discov. *2*, 16010. https://doi.org/10.1038/celldisc.2016.10.

Liu, Z., Cai, Y., Wang, Y., Nie, Y., Zhang, C., Xu, Y., Zhang, X., Lu, Y., Wang, Z., Poo, M., and Sun, Q. (2018). Cloning of macaque monkeys by somatic cell nuclear transfer. Cell *172*, 881–887.e7. https://doi.org/10.1016/j.cell.2018.01.020.

Liu, Z., Cai, Y., Liao, Z., Xu, Y., Wang, Y., Wang, Z., Jiang, X., Li, Y., Lu, Y., Nie, Y., et al. (2019). Cloning of a gene-edited macaque monkey by somatic cell nuclear transfer. Natl. Sci. Rev. *6*, 101–108. https://doi.org/10.1093/nsr/nwz003.

Lonfat, N., and Duboule, D. (2015). Structure, function and evolution of topologically associating domains (TADs) at HOX loci. FEBS Lett. *589*, 2869–2876. https://doi.org/10.1016/j.febslet.2015.04.024.

Mansfield, K. (2003). Marmoset models commonly used in biomedical research. Comp. Med. *53*, 383–392.

Matoba, S., and Zhang, Y. (2018). Somatic cell nuclear transfer reprogramming: mechanisms and applications. Cell Stem Cell *23*, 471–485. https://doi.org/10.1016/j.stem.2018.06.018.

Matoba, S., Liu, Y., Lu, F., Iwabuchi, K.A., Shen, L., Inoue, A., and Zhang, Y. (2014). Embryonic development following somatic cell nuclear transfer impeded by persisting histone methylation. Cell *159*, 884–895. https://doi.org/10.1016/j.cell.2014.09.055.

Matoba, S., Wang, H., Jiang, L., Lu, F., Iwabuchi, K.A., Wu, X., Inoue, K., Yang, L., Press, W., Lee, J.T., et al. (2018). Loss of H3K27me3 imprinting in somatic cell nuclear transfer embryos disrupts post-implantation development. Cell Stem Cell *23*, 343–354.e5. https://doi.org/10.1016/j.stem.2018.06.008.

Matoba, S., Kozuka, C., Miura, K., Inoue, K., Kumon, M., Hayashi, R., Ohhata, T., Ogura, A., and Inoue, A. (2022). Noncanonical imprinting sustains embryonic development and restrains placental overgrowth. Genes Dev. *36*, 483–494. https://doi.org/10.1101/gad.349390.122.

Matoba, S., Shikata, D., Shirai, F., Tatebe, T., Hirose, M., Nakata, A., Watanabe, N., Hasegawa, A., Ito, A., Yoshida, M., and Ogura, A. (2024). Reduction of H3K9 methylation by G9a inhibitors improves the development of mouse SCNT embryos. Stem Cell Rep. *19*, 906–921. https://doi.org/10.1016/j.stemcr.2024.04.003.

Miyamoto, K., Tajima, Y., Yoshida, K., Oikawa, M., Azuma, R., Allen, G.E., Tsujikawa, T., Tsukaguchi, T., Bradshaw, C.R., Jullien, J., et al. (2017). Reprogramming towards totipotency is greatly facilitated by synergistic effects of small molecules. Biol. Open *6*, 415–424. https://doi.org/10.1242/bio.023473.

Nishigaya, Y., Takase, S., Sumiya, T., Kikuzato, K., Sato, T., Niwa, H., Sato, S., Nakata, A., Sonoda, T., Hashimoto, N., et al. (2023). Discovery of novel substrate-competitive lysine methyltransferase G9a inhibitors as anticancer agents. J. Med. Chem. *66*, 4059–4085. https://doi.org/10.1021/acs.jmedchem.2c02059.

Ogonuki, N., Inoue, H., Matoba, S., Kurotaki, Y.K., Kassai, H., Abe, Y., Sasaki, E., Aiba, A., and Ogura, A. (2018). Oocyte-activating capacity of fresh and frozen–thawed spermatids in the common marmoset (Callithrix jacchus). Mol. Reprod. Dev. *85*, 376–386. https://doi.org/10.1002/mrd.22971.

Ogura, A., Matoba, S., and Inoue, K. (2021). Epigenetic abnormalities associated with somatic cell nuclear transfer. Reproduction *162*, F45–F58. https://doi.org/10.1530/REP-21-0013.

Sasaki, E., Hanazawa, K., Kurita, R., Akatsuka, A., Yoshizaki, T., Ishii, H., Tanioka, Y., Ohnishi, Y., Suemizu, H., Sugawara, A., et al. (2005). Establishment of novel embryonic stem cell lines derived from the common marmoset (*Callithrix jacchus*). Stem Cell. *23*, 1304–1313. https://doi.org/10.1634/stemcells.2004-0366.

Sasaki, E., Suemizu, H., Shimada, A., Hanazawa, K., Oiwa, R., Kamioka, M., Tomioka, I., Sotomaru, Y., Hirakawa, R., Eto, T., et al. (2009). Generation of transgenic non-human primates with germline transmission. Nature *459*, 523–527. https://doi.org/10.1038/nature08090.

Sato, K., and Sasaki, E. (2018). Genetic engineering in nonhuman primates for human disease modeling. J. Hum. Genet. *63*, 125–131. https://doi.org/10.1038/s10038-017-0351-5.

Sato, K., Oiwa, R., Kumita, W., Henry, R., Sakuma, T., Ito, R., Nozu, R., Inoue, T., Katano, I., Sato, K., et al. (2016). Generation of a nonhuman primate model of severe combined immunodeficiency using highly efficient genome editing. Cell Stem Cell *19*, 127–138. https://doi.org/10.1016/j.stem.2016.06.003.

Sato, K., Sasaguri, H., Kumita, W., Sakuma, T., Morioka, T., Nagata, K., Inoue, T., Kurotaki, Y., Mihira, N., Tagami, M., et al. (2024). Production of a heterozygous exon skipping model of common marmosets using gene-editing technology. Lab Anim. (NY) *53*, 244–251. https://doi.org/10.1038/s41684-024-01424-0.

Sotomaru, Y., Hirakawa, R., Shimada, A., Shiozawa, S., Sugawara, A., Oiwa, R., Nobukiyo, A., Okano, H., Tamaoki, N., Nomura, T., et al. (2009). Preimplantation development of somatic cell cloned embryos in the common marmoset (*Callithrix jacchus*). Clon Stem Cell *11*, 575–583. https://doi.org/10.1089/clo.2009.0005.

Sugawara, A., Goto, K., Sotomaru, Y., Sofuni, T., and Ito, T. (2006). Current status of chromosomal abnormalities in mouse embryonic stem cell lines used in Japan. Comp. Med. *56*, 31–34.

Takahashi, T., Hanazawa, K., Inoue, T., Sato, K., Sedohara, A., Okahara, J., Suemizu, H., Yagihashi, C., Yamamoto, M., Eto, T., et al. (2014). Birth of healthy offspring following ICSI in in vitro-matured common marmoset (Callithrix jacchus) oocytes. PLoS One *9*, e95560. https://doi.org/10.1371/journal.pone.0095560.

Takase, S., Hiroyama, T., Shirai, F., Maemoto, Y., Nakata, A., Arata, M., Matsuoka, S., Sonoda, T., Niwa, H., Sato, S., et al. (2023). A specific G9a inhibitor unveils BGLT3 lncRNA as a universal mediator of chemically induced fetal globin gene expression. Nat. Commun. *14*, 23. https://doi.org/10.1038/s41467-022-35404-0.

Terashita, Y., Wakayama, S., Yamagata, K., Li, C., Sato, E., and Wakayama, T. (2012). Latrunculin A can improve the birth rate of cloned mice and simplify the nuclear transfer protocol by gently inhibiting actin polymerization. Biol. Reprod. *86*, 180. https://doi.org/10.1095/biolreprod.111.098764.

Van Thuan, N., Bui, H.-T., Kim, J.-H., Hikichi, T., Wakayama, S., Kishigami, S., Mizutani, E., and Wakayama, T. (2009). The histone deacetylase inhibitor scriptaid enhances nascent mRNA production and rescues full-term development in cloned inbred

mice. Reproduction *138*, 309–317. https://doi.org/10.1530/REP-08-0299.

Wang, L.Y., Li, Z.K., Wang, L.B., Liu, C., Sun, X.H., Feng, G.H., Wang, J.Q., Li, Y.F., Qiao, L.Y., Nie, H., et al. (2020). Overcoming intrinsic H3K27me3 imprinting barriers improves post-implantation development after somatic cell nuclear transfer. Cell Stem Cell *27*, 315–325.e5. https://doi.org/10.1016/j.stem.2020.05.014.

Xu, R., Zhu, Q., Zhao, Y., Chen, M., Yang, L., Shen, S., Yang, G., Shi, Z., Zhang, X., Shi, Q., et al. (2023). Unreprogrammed H3K9me3 prevents minor zygotic genome activation and lineage commitment in SCNT embryos. Nat. Commun. *14*, 4807. https://doi.org/10.1038/s41467-023-40496-3.

Zhou, X., Liao, W.-J., Liao, J.-M., Liao, P., and Lu, H. (2015). Ribosomal proteins: functions beyond the ribosome. J. Mol. Cell Biol. *7*, 92–104. https://doi.org/10.1093/jmcb/mjv014.

Stem Cell Reports, Volume *20*

Supplemental Information

Derivation of embryonic stem cells from cloned blastocysts using improved somatic cell nuclear transfer in common marmosets

Shogo Matoba, Yoko Kurotaki, Satoshi Funaya, Yuko Yamada, Narumi Ogonuki, Haruka Shinohara, Masafumi Yamamoto, Nao Yoneda, Takaya Homma, Yuichiro Higuchi, Erika Sasaki, and Atsuo Ogura

Supplemental Figures

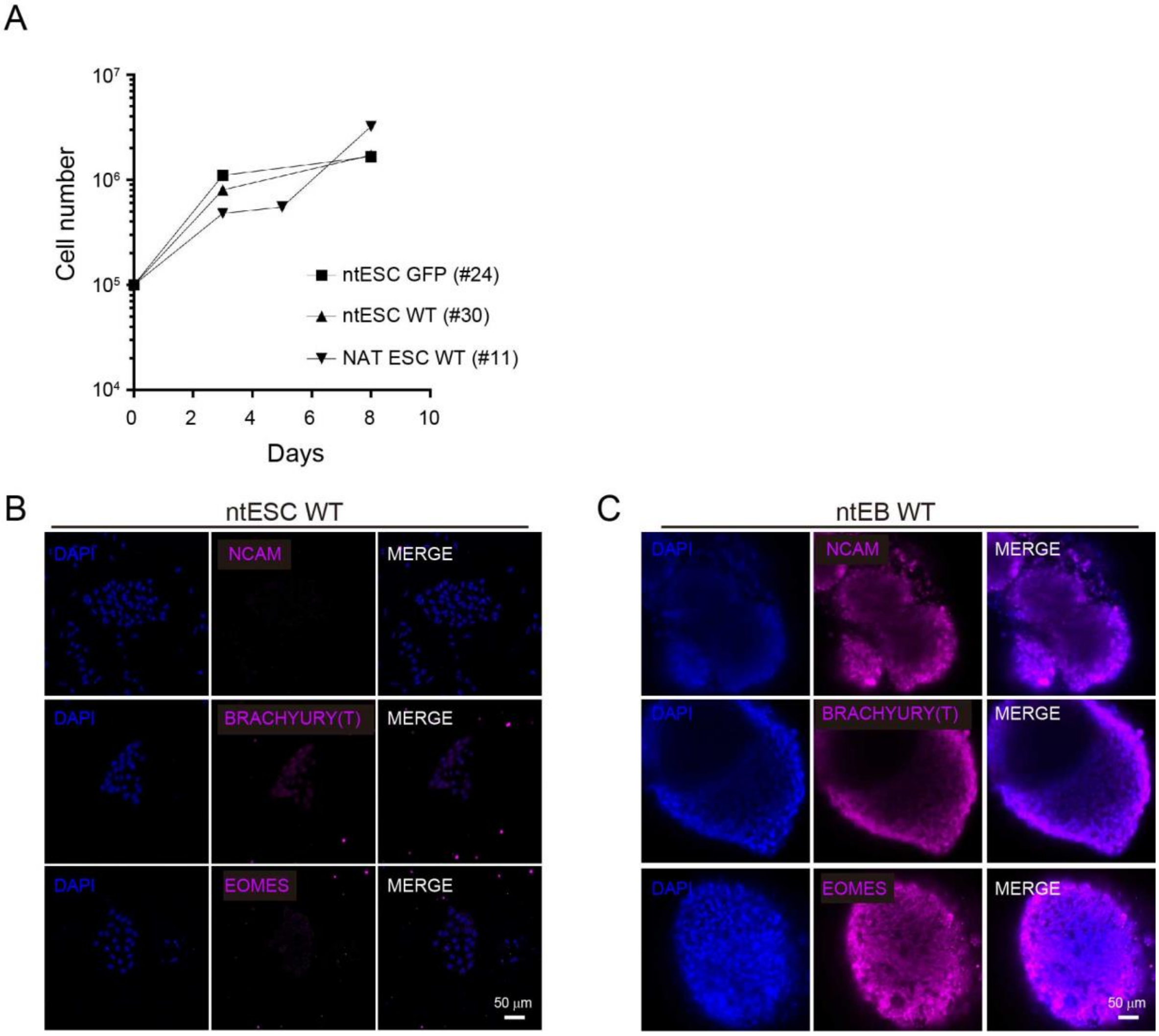

Figure S1. Proliferation and in vitro differentiation of ntESCs to form ntEBs, related to Figure 5

(A) Proliferation of ESCs and ntESCs. (B-C) Immunostaining (B) ntESCs and (C) ntEBs of WT #61 line for markers of three germ layers.

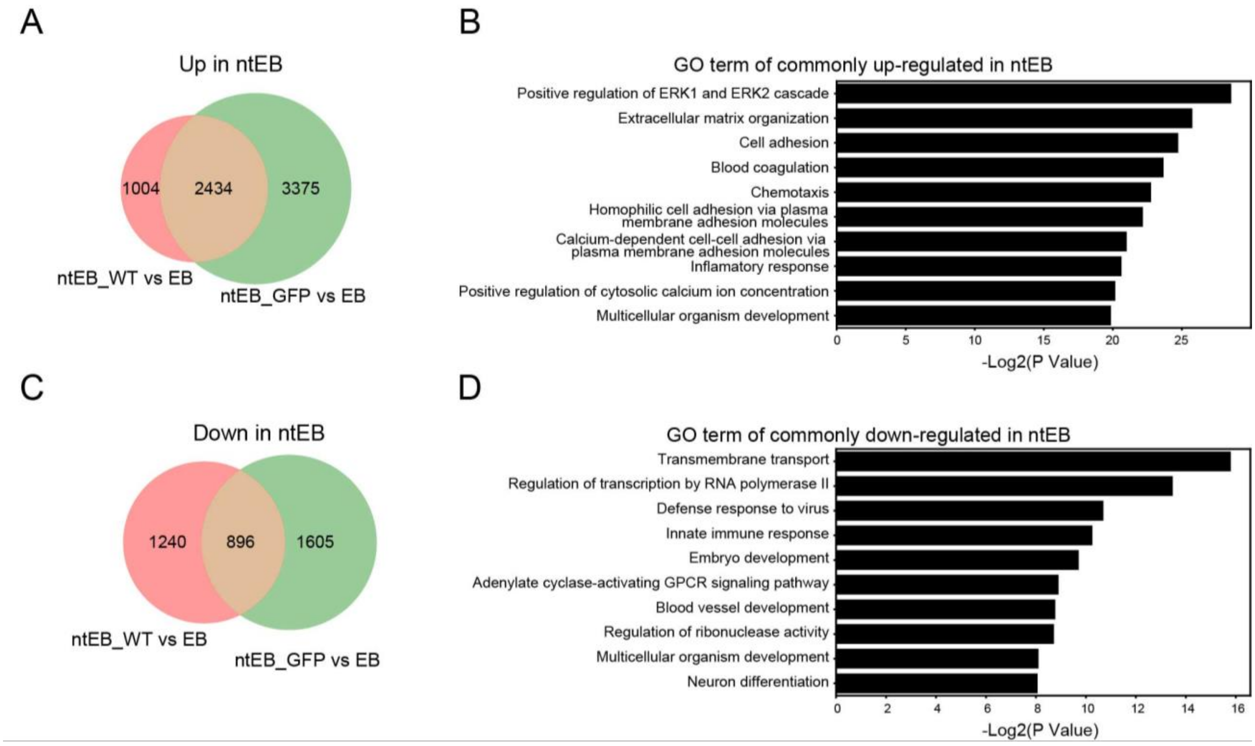

Figure S2. Abnormally expressed genes in ntEB, related to Figure 6
(A) Venn diagram showing commonly upregulated genes in ntEB.
(B) GO term enriched in the commonly upregulated genes in ntEB.
(C) Venn diagram showing commonly downregulated genes in ntEB.
(D) GO term enriched in the commonly downregulated genes in ntEB.

Supplemental Tables

Table S1. List of established ntESCs in this study, related to Figure 3

Name of ESC	Genotype	Manipulation	Sex of donor cell	Karyotype analysis		
				Passage number	Mode number	Karyotype
ntESC WT #30	WT	SCNT	XY	6	46 (84%)	Normal 46, XY [8/8]
ntESC WT #53	WT	SCNT	XY	11	46 (90%)	Normal 46, XY [5/5]
ntESC WT #61	WT	SCNT	XY	10	46 (90%)	Normal 46, XY [8/8]
ntESC GFP #24	CAG-GFP	SCNT	XX	5	46 (94%)	Normal 46, XX [5/5]
ntESC GFP #50	CAG-GFP	SCNT	XX	6	46 (89%)	Normal 46, XX [5/5]
ntESC GFP #54	CAG-GFP	SCNT	XX	6	46 (98%)	Normal 46, XX [5/5]
				11	46 (98%)	Abnormal 46, XX, t(7p;15q) [5/5]
ntESC GFP #55	CAG-GFP	SCNT	XX	7	46 (80%)	Normal 46, XX [5/5]
ESC NAT #11	WT	NAT	XY	7	46 (58%)	Normal 46, XY [11/11]
ESC NAT #13	WT	NAT	XX	18	46 (88%)	Normal 46, XX [7/7]
ESC NAT #37	WT	NAT	XY	7	46 (86%)	Normal 46, XY [6/6]
ESC IVF #15	WT	IVF	XX	14	46 (86%)	Normal 46, XX [7/7]
ESC IVF #17	WT	IVF	XY	21	46 (66%)	Normal 46 XY [6/6]

*NAT and IVF derived ESCs were reported in Kishimoto et al. 2021.

Table S2. RNA-sequencing data comparing control ESC and ntESC, related to Figures 4, 5 and 6 (see Excel file)

Table S3. RNA-sequencing data comparing control EB and ntEB, related to Figures 5 and S2 (see Excel file).

Table S4. List of primers used for microsatellite marker analysis, related to Figure 3

Type	Name of primer	Dye	Sequence
Dye-labeled primer	2463P-TH	FAM	GCACAGGCAGATTCAAGACAACTC
	CJ060	PET	TGCTCTAGAGGTTCCACTCTG
	CJ077	PET	ATTCCATTCTGGGCAGCAAG
	CJ081	VIC	TTCCCCTCTCTTTCAGACACA
	CJ103	NED	CCCTTTCCTGCTAATTCACAGAAG
	CJ187	FAM	TGGAAGAACTTTCTGCCAAACC
	CJ003	NED	AGATGTGGCAGTTGTCTTGG
	CJ083	VIC	TTGTACCCTTTTGCTTGCAG
	CJ091	FAM	CCTGCACCCGTAAATAGGTTC
	CJ146	PET	CTTAATTCTGCCACAGTAGCAC
	CjX	FAM	CCAGCTACTCATGAAGTTGAG
	CjY	VIC	CATTGTGCCACTGCAGAATTA
Non-labeled primer	2463P-TH-NL	None	CCAAGACCTCAGGGAGGTAGTAGG
	CJ060-NL	None	GGCATGTTACCTAACCTCTCTG
	CJ077-NL	None	CCTCCCATACTACAGATGAGGA
	CJ081-NL	None	CACCTCCTCTTCAAGTAAACACC
	CJ103-NL	None	CTGGGTAACAAGAGTGAAACTCC
	CJ187-NL	None	GCTTGTTCAGGCAGACTGAC
	CJ003-NL	None	TCTCTGCCATAGTGACCTCT
	CJ083-NL	None	TTCCTTCTTTTGGGGAGTGT
	CJ091-NL	None	CATCCTGGGCAACAAGAGTG
	CJ146-NL	None	GAGAGTCCCTAAATGCAAGGA
	CjX-NL	None	CTCTTCATATTCTTATGATGTGTGC
	CjY-NL	None	GAAGGTAATCCATTTCACTGAGC

Table S5. List of antibodies used for immunofluorescence staining, related to Figures 1 and 4

Antibody name	Dilution	Source animal	Catalog number	Clone ID	Company
anti-H3K9me3	1:500	Rabbit	ab8898		Abcam
anti-NANOG	1:200	Mouse	4893	1E6C4	Cell Signaling Technology
anti-CDX2	1:400	Rabbit	3977		Cell Signaling Technology
anti-GFP	1:100	Rabbit	ab183734		Abcam
anti-OCT3/4	1:50	Mouse	sc-5279	C-10	Santa Cruz Biotechnology
anti-SOX2	1:200	Mouse	4900	L1D6A2	Cell Signaling Technology
anti-LIN28A	1:200	Rabbit	3978		Cell Signaling Technology
anti-SSEA4	1:200	Mouse	sc-21704	813-70	Santa Cruz Biotechnology
anti-TRA-1-60	1:100	Mouse	MAB4360	TRA-1-60	Thermo Fisher Scientific
anti-TRA-1-81	1:100	Mouse	MAB4381	TRA-1-81	Merck Millipore
anti-UTF1	1:100	Mouse	MAB4337	5G10.2	Merck Millipore
anti-NCAM-L1	1:200	Rabbit	89861	D5N9S	Cell Signaling Technology
anti-BRACHYURY (T)	1:200	Rabbit	ab20680		Abcam
anti-EOMES	1:100	Rabbit	ab23345		Abcam

Supplemental methods

Oocyte collection and in vitro maturation

Oocyte retrieval was performed as previously described (Kurotaki and Sasaki, 2017; Sato et al., 2016; Takahashi et al., 2014; Tomioka et al., 2012). To synchronize the estrous cycle, female common marmosets were injected intramuscularly with 0.8 µg of prostaglandin F2α (MSD Animal Health, USA) to induce luteolysis. The next day, serum progesterone concentration was confirmed to have decreased, and ovarian stimulation was initiated. Recombinant follicle-stimulating hormone (rFSH; 25 IU/head; Gonal-f 150IU, Merck, Germany) was administered intramuscularly every second day for 9 days. On the evening of day 10, human chorionic gonadotropin (hCG; 75 IU/head; ASKA Pharmaceutical, Japan) was injected. Oocyte pick-up (OPU) was performed by laparotomy 18 hours after hCG injection.

For pre-anesthesia, animals were given 0.15 ml of an MMB cocktail (medetomidine 0.04 mg/kg, midazolam 0.4 mg/kg, butorphanol 0.4 mg/kg and saline at a 1:2:2:5 ratio: Dexmedetomidine, Sandoz, Japan; Midazolam, Teva Takeda Pharma, Japan; Vetorphale, Meiji Seika Pharma, Japan; and saline, Otsuka Pharmaceutical, Japan). To prevent infection and dehydration, ampicillin (15 mg/kg, Fujita Pharmaceutical, Japan) and an electrolyte solution (KN No. 1, Otsuka Pharmaceutical) were administered intramuscularly. Anesthesia was maintained using 1.0%–3.0% isoflurane with a face mask. After oocyte retrieval, the animal's abdomen was sutured, and the animal was rested for at least 2 months before the next OPU. Atipamezole (0.20 mg/kg, Nippon Zenyaku Kogyo, Japan) was administered for recovery. For post-operative care, ketoprofen (1.2 mg/kg, Nissin Pharmaceutical, Japan) and ampicillin (15 mg/kg) were administered daily for three days.

Oocytes were aspirated from ovarian follicles using a 25-gauge needle and cultured in porcine oocyte medium (POM) (IFP1010P, Research Institute for the Functional Peptides, Japan). The retrieved GV oocytes were matured in vitro in POM supplemented with 0.15 IU/mL hFSH, 10 IU/mL hCG and 5% FBS for 24 hours under controlled conditions of 37.5 °C in a humidified atmosphere containing 5% CO_2 and 5% O_2 in air. Cumulus cells were removed by hyaluronidase treatment, and MII oocytes were selected for SCNT.

Preparation of donor cells

Somatic cells used for nuclear transfer included cumulus cells and fibroblasts. Cumulus cells were collected by enzymatic dissociation of cumulus-oocyte complexes obtained from in vitro-matured oocytes derived from WT female marmosets.

Fibroblasts were isolated from ear skin biopsies of WT and GFP-transgenic marmosets using standard tissue digestion protocols. Cells were cultured for several days and frozen at early passages (P3–P6). For SCNT, fibroblasts were thawed and seeded into 60-mm culture dishes (3010-060, AGC Techno Glass, Japan) 7 days prior to use. After reaching confluency, cells were maintained for several days to synchronize in the G1/G0 phase.

Immunofluorescence staining

SCNT embryos, ntESCs, or ntEBs were fixed with 4% PFA in PBS for 30 minutes at room temperature, washed with PBS, and permeabilized with 0.5% Triton X-100 for 10 minutes. After blocking with 5% FBS in PBS for 30 minutes, embryos or cells were incubated overnight at 4 °C with primary antibodies (Table S5). After washing, samples were incubated with Alexa Fluor 555-conjugated donkey anti-rabbit IgG, Alexa Fluor 647-conjugated donkey anti-mouse, or anti-rabbit IgG secondary antibodies (Thermo Fisher Scientific) for 30 minutes at room temperature. Nuclei were stained with DAPI (P36931, Invitrogen, USA) or Hoechst 33342 (346-07951, DOJINDO). Samples were visualized using an IX71 fluorescence microscope (Olympus, Japan) or LSM880 confocal microscope (Zeiss, Germany).

Supplemental references

Kurotaki, Y., and Sasaki, E. (2017). Practical reproductive techniques for the common marmoset. J. Mamm. Ova Res. *34*, 3–12. 10.1274/032.034.0103.

Sato, K., Oiwa, R., Kumita, W., Henry, R., Sakuma, T., Ito, R., Nozu, R., Inoue, T., Katano, I., Sato, K., et al. (2016). Generation of a nonhuman primate model of severe combined immunodeficiency using highly efficient genome editing. Cell Stem Cell *19*, 127–138. 10.1016/j.stem.2016.06.003.

Takahashi, T., Hanazawa, K., Inoue, T., Sato, K., Sedohara, A., Okahara, J., Suemizu, H., Yagihashi, C., Yamamoto, M., Eto, T., et al. (2014). Birth of healthy offspring following ICSI in in vitro-matured common marmoset (Callithrix jacchus) oocytes. PLoS One *9*, e95560. 10.1371/journal.pone.0095560.

Tomioka, I., Takahashi, T., Shimada, A., Yoshioka, K., and Sasaki, E. (2012). Birth of common marmoset (Callithrix jacchus) offspring derived from in vitro-matured oocytes in chemically defined medium. Theriogenology *78*, 1487–1493. 10.1016/j.theriogenology.2012.06.024.
